# An accurate method for identifying recent recombinants from unaligned sequences

**DOI:** 10.1093/bioinformatics/btac012

**Published:** 2022-01-13

**Authors:** Qian Feng, Kathryn E Tiedje, Shazia Ruybal-Pesántez, Gerry Tonkin-Hill, Michael F Duffy, Karen P Day, Heejung Shim, Yao-Ban Chan

**Affiliations:** Melbourne Integrative Genomics/School of Mathematics and Statistics, The University of Melbourne, Melbourne, VIC 3010, Australia; School of BioSciences, The University of Melbourne, Bio21 Molecular Science and Biotechnology Institute, Melbourne, VIC 3010, Australia; Department of Microbiology and Immunology, The University of Melbourne, at the Peter Doherty Institute for Infection and Immunity and Bio21 Molecular Science and Biotechnology Institute, Melbourne, VIC 3000, Australia; School of BioSciences, The University of Melbourne, Bio21 Molecular Science and Biotechnology Institute, Melbourne, VIC 3010, Australia; Population Health and Immunity Division, Walter and Eliza Hall Institute of Medical Research, Melbourne, VIC 3052, Australia; Department of Medical Biology, The University of Melbourne, Melbourne, VIC 3010, Australia; Burnet Institute, Melbourne, VIC 3004, Australia; School of BioSciences, The University of Melbourne, Bio21 Molecular Science and Biotechnology Institute, Melbourne, VIC 3010, Australia; Bioinformatics Division, Walter and Eliza Hall Institute of Medical Research, Melbourne, VIC 3052, Australia; Parasites and Microbes, Wellcome Sanger Institute, Wellcome Genome Campus, Hinxton CB10 1SA, UK; Peter Doherty Institute for Infection and Immunity, Melbourne, VIC 3004, Australia; School of BioSciences, The University of Melbourne, Bio21 Molecular Science and Biotechnology Institute, Melbourne, VIC 3010, Australia; Department of Microbiology and Immunology, The University of Melbourne, at the Peter Doherty Institute for Infection and Immunity and Bio21 Molecular Science and Biotechnology Institute, Melbourne, VIC 3000, Australia; Melbourne Integrative Genomics/School of Mathematics and Statistics, The University of Melbourne, Melbourne, VIC 3010, Australia; Melbourne Integrative Genomics/School of Mathematics and Statistics, The University of Melbourne, Melbourne, VIC 3010, Australia

## Abstract

**Motivation:**

Recombination is a fundamental process in molecular evolution, and the identification of recombinant sequences is thus of major interest. However, current methods for detecting recombinants are primarily designed for aligned sequences. Thus, they struggle with analyses of highly diverse genes, such as the *var* genes of the malaria parasite *Plasmodium falciparum*, which are known to diversify primarily through recombination.

**Results:**

We introduce an algorithm to detect recent recombinant sequences from a dataset without a full multiple alignment. Our algorithm can handle thousands of gene-length sequences without the need for a reference panel. We demonstrate the accuracy of our algorithm through extensive numerical simulations; in particular, it maintains its effectiveness in the presence of insertions and deletions. We apply our algorithm to a dataset of 17 335 DBL*α* types in *var* genes from Ghana, observing that sequences belonging to the same ups group or domain subclass recombine amongst themselves more frequently, and that non-recombinant DBL*α* types are more conserved than recombinant ones.

**Availability and implementation:**

Source code is freely available at https://github.com/qianfeng2/detREC_program.

**Supplementary information:**

Supplementary data are available at *Bioinformatics* online.

## 1 Introduction

Recombination, the exchange of genetic materials between two molecular sequences, is a fundamental evolutionary process in viruses, prokaryotes, eukaryotes and even between kingdoms. The biological mechanisms of recombination, which differ across different species, lead to the creation of novel ‘mosaic’ sequences in which different regions have distinct evolutionary histories.

In population genetics, recombination plays a central role in shaping the patterns of linkage disequilibrium, and thus recombination identification is of importance for estimating recombination rates, quantitative trait loci and association studies (Drysdale *et al.*, 2000; Li and Stephens, 2003). Recombination also explains a considerable amount of the genetic diversity of human pathogens (Gibbs *et al.*, 2001; Holmes *et al.*, 1999; Robertson *et al.*, 1995), such as the malaria parasite *Plasmodium falciparum* (Claessens *et al.*, 2014; Jiang *et al.*, 2011) or protozoan parasites (Weatherly *et al.*, 2016). It plays a central role for parasites to escape from host immune pressures, or adapt to the effects of antiparasitic drugs. Characterization of recombination events in these pathogens would aid in the understanding of these evolutionary mechanisms.

Many methods have been developed for identifying recombination events and/or recombinants (e.g. Auton and McVean, 2007; Boni *et al.*, 2007, see Lemey *et al.*, 2009 for a review; Kosakovsky Pond *et al.*, 2006; Martin and Rybicki, 2000; Posada and Crandall, 2001). They can be roughly characterized into four paradigms:

1. Distance-based methods (Buendia and Narasimhan, 2007; Huber *et al.*, 2004; Siepel *et al.*, 1995) look for inversions of distance patterns among the sequences. They usually use a sliding-window approach to estimate distances and are generally computationally efficient.
2. Phylogenetic methods (Hein, 1990; Holmes *et al.*, 1999; Martin and Rybicki, 2000) look for discordant topologies in adjacent sequence segments, which is taken as a sign of recombination.
3. Compatibility methods (Jakobsen and Easteal, 1996) test for phylogenetic incongruence on a site-by-site basis.
4. Substitution distribution-based methods (Boni *et al.*, 2007; Posada and Crandall, 2001; Smith, 1992) use a test statistic to examine adjacent sequence segments for signals of recombination.

Nearly all available methods require a multiple sequence alignment; this is commonly available for population genetic datasets which have relatively low intra-population diversity, but may be unreliable for datasets with higher diversity. Likewise, many of the most commonly used methods, such as RDP (Martin and Rybicki, 2000) or 3SEQ (Boni *et al.*, 2007), are triplet-based; that is, they test for recombination signals in each possible triplet of sequences, which can become slow as modern-day datasets grow larger and larger. Finally, some (though not all) methods (e.g. Siepel *et al.*, 1995) require a reference panel of known non-recombinant sequences, which potential recombinants can be compared against. We aim to develop a method which works directly on sequences without requiring a full multiple sequence alignment or a reference panel, and is fast enough to be practical for large datasets.

We focus on the specific application of detecting recombinants in the *var* genes of *P.falciparum*. These genes express the *P.falciparum* erythrocyte membrane protein 1 (PfEMP1), which is the main target of the human immune response to the blood stages of infection. The *var* genes are a large and diverse gene family (up to 60 copies per genome), and high levels of diversity in the *var* genes have been observed in a single parasite genome, as well as small local populations (Chen *et al.*, 2011; Day *et al.*, 2017; Rask *et al.*, 2010; Ruybal-Pesántez *et al.*, 2017). This diversity is driven primarily by homologous recombination (Claessens *et al.*, 2014), and so an accurate identification of *var* recombinants is critical to understanding the evolution of the system.

We focus on the DBL*α* domain, which is the only domain encoded by all (but one) members of the *var* multigene family. This domain has been found to be immunogenic (Tessema *et al.*, 2019) and is crucial to understanding acquired immunity and potential for vaccination (Sherman, 2011). Unfortunately, the DBL*α* domain is highly variable in terms of both length and sequence composition, with datasets (Tonkin-Hill *et al.*, 2021) containing tens of thousands of disparate sequences. Under these conditions, multiple sequence alignments constructed from these datasets are very unreliable, and a phylogenetic tree is not an appropriate representation of their evolutionary history due to frequent recombination. Thus, it is difficult to reconstruct an explicit evolutionary history of the DBL*α* domain.

The first systematic attempt to map out recombination in *var* genes was performed by Zilversmit *et al.* (2013), who developed a method based on a jumping hidden Markov model (JHMM) to align a sequence to its nearest relations in a reference dataset, allowing jumps between sequences which represent recombination events. They used this method to ‘paint’ each sequence according its nearest relations. However, this method does not identify the recombinant sequences themselves, only recombination events. An explicit identification of recombinants and non-recombinants would enable direct comparison between them, helping to determine the effect of recombination on the structure and function of the gene.

Because each sequence is considered individually, the JHMM is limited to the detection of ‘recent’ recombination events; that is, recombinations whose signal can be found only in one sequence in the dataset. In contrast, a single more ancient recombination may leave traces in multiple sequences, hindering the ability to detect them. It is thus an unavoidable consequence that any method based on the information provided by the JHMM is limited to the detection of recent recombinants, i.e. the descendants of recent recombinations.

In this article, we develop a new method to identify recent recombinants in a large dataset of sequences, that does not require a multiple sequence alignment. This method exploits the information produced by the JHMM method, combining it with a distance-based comparison to identify recombinants. Extensive simulations confirm the accuracy and applicability of our method, in particular in the context of sequences with insertions and deletions. We also show that our method is more accurate than many currently used methods. Finally, we apply our method to a large dataset of DBL*α* sequences, producing several new biological results concerning the patterns of recombination in this domain.

## 2 Materials and methods

We propose a novel method to detect recombinant sequences in a set of protein or DNA sequences for which a full multiple alignment is difficult to construct or unreliable. It takes as input a set of homologous sequences, and outputs the sequences that are identified as recombinant, their putative parents, and the corresponding breakpoints.

**Fig. 1.**
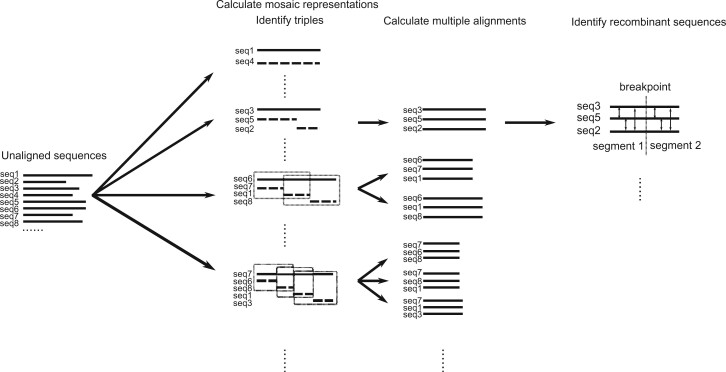
A schematic of the algorithm. From an input set of unaligned sequences, we first use the JHMM method to represent each sequence as a mosaic of other sequences. Next, we identify triples of segments, consisting of a recombinant segment and its two parents, and complete their alignment with the MAFFT algorithm. Finally, we identify the recombinant in each triple using a distance-based approach

See Figure 1 for a graphical overview of our method. It consists of the following steps:

1. We apply the JHMM method of Zilversmit *et al.* (2013) to represent each sequence as a ‘mosaic’ of segments from other sequences in the dataset.
2. We identify ‘recombinant triples’ which contain a recombinant segment and its two parents. The mosaic representations provide pairwise alignments for each of these triples, which we then complete to three-way alignments with the MAFFT algorithm (Katoh and Frith, 2012).
3. Using a distance-based approach, we identify the recombinant sequence in each triple.

Note that, extant sequences are identified as the ‘parents’ of the recombinant; more accurately, we identify the descendants of the ancestral sequences which were the parents of the recombination.

We discuss each step in detail in the following sections.

### 2.1 Calculating mosaic representations

We first use the jumping hidden Markov model of Zilversmit *et al.* (2013). In this model, each character in a ‘target’ sequence is considered to be a copy from a character in a sequence in a reference set (‘source’ sequences). The hidden state of the Markov model is the (position of the) character which is copied. The copy may be imperfect, representing mutation. After a character is copied, the next character in the target sequence is usually copied from the next character in the same source sequence. However, with small probabilities:

- the source character may switch to any character in any position in another sequence, representing recombination;
- the model switches to an ‘insertion’ state, where the target character is chosen randomly and the source character does not move;
- the model switches to a ‘deletion’ state, where the source character moves forward without being copied.

If the model is in an insertion or deletion state, it continues in this state until (with a small probability per character) we return to copying characters from the current source sequence.

We first estimate the parameters of the model, following Tonkin-Hill *et al.* (2021). The parameters are the probabilities of gap initiation *δ*, gap extension *ϵ* and recombination (source switching) *ρ*. We first set *ρ* to zero, and compute maximum likelihood estimates for *δ* and *ϵ* with the Baum-Welch algorithm (see Rabiner, 1989). We then calculate the composite likelihood of all sequences for all values of *ρ* over the interval [0,0.1] under the estimated $\hat{\delta }$ and $\hat{\epsilon }$, and choose the value of *ρ* which maximizes this likelihood as our estimate $\hat{\rho }$.

Finally, we calculate the Viterbi path for each target sequence to find the most probable sequence of hidden states (copied characters, insertions and deletions). The result is a ‘mosaic’ alignment for each sequence to a series of segments from the other sequences in the dataset. An example of this can be seen in Figure 2A in Zilversmit *et al.* (2013).

**Fig. 2.**
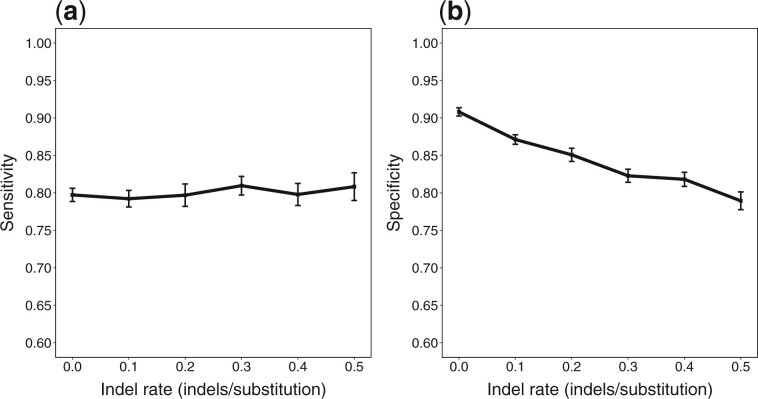
Mean sensitivity and specificity (with 95% confidence intervals) for varying indel rate

For large-scale datasets, training the JHMM model is a significant bottleneck for our method. We again follow Tonkin-Hill *et al.* (2021), and use the Viterbi training algorithm (Rodríguez and Torres, 2003) in place of the Baum-Welch to estimate *δ* and *ϵ*, and calculate the composite likelihood over 1000 randomly selected sequences to estimate *ρ*. This allows us to analyze large datasets (such as the DBL*α* dataset in Section 3.2) in a practical timeframe with only a small loss in accuracy.

### 2.2 Identifying recombinant triples and calculating multiple sequence alignments

For each breakpoint in each sequence, we identify the triple of the target sequence and the two sequences which contain the source segments before and after the breakpoint as a ‘recombinant triple’, that is, the two parents and the child of a recombination. This results in a list of recombinant triples, some of which may refer to the same recombination event. The JHMM method only provides a pairwise alignment of each target segment to one source segment. We take these pairwise alignments and add the corresponding segment from the remaining source sequence in the triple, using the MAFFT algorithm (Katoh and Frith, 2012). For each triple, this results in a multiple alignment of the segments surrounding the breakpoint. See Supplementary Figure S16 for an overview of this process.

Note that, we require a sufficient sequence length on either side of the breakpoint to calculate distances accurately. Moreover, we observe in practice that short source segments resulting from the JHMM method tend to be artifacts of the method, rather than representing multiple consecutive recombinations. To address this, we exclude triples for which the aligned segment on either side of the breakpoint has length less than 10, which we found to be a suitable threshold in practice.

### 2.3 Identifying recombinant sequences

We now apply the well-known principle (Boni *et al.*, 2007; Posada and Crandall, 2001; Smith, 1992) that two non-recombinant sequences will have a similar evolutionary distance all along the sequence; that is, the distance between the two sequences does not change before and after a recombination breakpoint in a third sequence. Conversely, the distance between a recombinant sequence and another sequence does change at a breakpoint. Using a distance-based method here allows us to avoid an expensive tree or network inference step and thus scale our method to many sequences.

We calculate, for each recombinant triple {*a*, *b*, *c*}, the evolutionary distance between each pair of segments before and after the breakpoint. We use here the BLOSUM62 distance (Henikoff and Henikoff, 1992) for amino acids and Hamming (mismatch) distance for DNA sequences (these could in principle be substituted by a large variety of ways to calculate evolutionary distance). We denote these distances by *D*_1_ and *D*_2_ for the first (pre-breakpoint) and second (post-breakpoint) segment, respectively. The pair with the smallest absolute difference in distance before and after the breakpoint are inferred to be the two non-recombinant sequences, while the third is inferred to be recombinant. Formally, we have

$$\text{recombinant}=\{a,b,c\}\setminus {\text{argmin}}_{\left\{x,y\}\subset \{a,b,c\right\}}|D_{1}(x,y)-D_{2}(x,y)|.$$

This method identifies one recombinant from each recombinant triple; note that one recombination may generate one or more triples, but the identified recombinant from each of these triples should be the same. We apply this to all triples identified above, generating a list of recombinants in the entire dataset and their putative parents.

### 2.4 Calculating support values

In addition to identifying recombinant sequences, we can also measure the uncertainty in our identification by using bootstrapping. For each multiple alignment of a triple, we resample characters in the alignment (columns) within each segment, with replacement. This provides us with a resampled alignment, and we generate 100 replicates per triple. We then run our distance-based method to identify the recombinant for each replicate. The proportion of replicates which infer the same recombinant as the original alignment is the support value of this detection. The larger the support value, the more certain we are of the detection.

## 3 Results

### 3.1 Simulations

We conducted extensive simulations to evaluate the effectiveness of our method. Our simulation protocol is as follows:

1. Simulate a tree (genealogy) under the coalescent (without recombination) using msprime (Kelleher *et al.*, 2016).
2. Evolve amino acid sequences from a common ancestor along the tree using Pyvolve (Spielman and Wilke, 2015). If insertions and/or deletions are required, we use INDELible (Fletcher and Yang, 2009) instead.
3. Generate recombinant sequences from two or more randomly chosen sequences in the dataset, with breakpoints chosen uniformly at random along the genome. The parent sequences are removed from the dataset.

This simulation produces a dataset which can be clearly separated into recombinants and non-recombinants. Manually performing the recombination step guarantees that we have only recent recombinants, which our method is designed to detect. Moreover, the non-recombinants are guaranteed to have no ancient recombination events in their history. Note that, while we do not evolve our sequences further after recombination, we do remove the parents from the dataset, which produces a similar effect: their nearest extant relations in the dataset are evolutionarily separated from the recombinant sequence. In our simulations, we simulate both equal-length sequences (no indels), and unequal-length sequences with indel events, generating unaligned input.

There are a wide variety of parameters which could potentially affect the performance of the method. We vary the proportion of recombinant sequences in the dataset; the number of recombinations per recombinant; the number of sequences in the dataset; the sequence length; the mutation rate; and the substitution model. For simulations with insertions and deletions, we also vary indel rate and size. To keep our simulations tractable, we only vary one parameter at a time, keeping the remainder fixed at default values (Supplementary Tables S2 and S3). For each parameter combination, we simulate 100 datasets and run our method on each dataset in turn.

Our results are shown in Supplementary Section S2. In summary, we find that the method enjoys good performance, with most parameter settings offering both sensitivity and specificity above 70% (and often much higher). For the simulations without indels, we find that sensitivity increases with the number of recombinations, sequence length and mutation rate, while staying stable with respect to the other parameters. Specificity decreases (usually slightly) as the proportion of recombinant sequences, number of recombinations, sequence length and mutation rate increase.

An important feature of our method is its ability to accept unaligned sequences as input. When we include indels in the generating process, we can see (Fig. 2) that both sensitivity and specificity remain relatively unaffected, with a moderate decline in specificity as indel rate increases. This indicates that our method is robust to indels even when the indel rate is large.

**Fig. 3.**
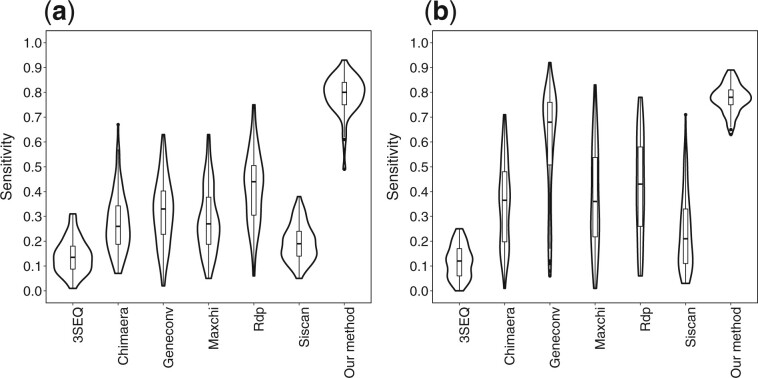
Distribution of sensitivity (for matched specificity) for different recombinant detection methods on simulated datasets with (left) and without (right) indel events

We also compared our method with a number of popular recombinant detection methods, after aligning the simulated sequences. We note that these methods only accept aligned sequences, making a direct comparison potentially biased one way or the other (depending on whether the sequences have indels or not). Despite this, we can see (Fig. 3) that our method enjoys the highest sensitivity overall when we matched the specificity of other methods to that of our method, whether or not indels are included in the sequences. For more details, see Supplementary Section S2.2.

Finally, we studied the distributions of the support values for true and false detections, and the accuracy of the JHMM methods in our simulations (Supplementary Sections S2.3 and S2.4).

### 3.2 Analysis of DBL*α* sequences from a cross-sectional study in Ghana

Population genetic studies of *var* genes have focused on sequencing the DBL*α* domain, since nearly all *var* genes encode a single DBL*α* domain. We applied our method to detect recombinants and breakpoints in a dataset of DBL*α* sequences collected from individuals with microscopically confirmed *P.falciparum* infections (*isolates*) living in the Bongo District, in the Upper East region of northern Ghana (GenBank BioProject Number: PRJNA396962) (He *et al.*, 2018; Pilosof *et al.*, 2019). This dataset consists of 35 591 previously published DBL*α* sequences collected from 161 isolates, which were clustered into 17 335 representative DBL*α* ‘types’ of average length 125aa (s.d. 8.4aa). Of these, we detected 14 801 (85.4%) to be recombinant. See Supplementary Section S3.1 for more details.

#### 3.2.1 DBLα sequences from the same ups group recombine more frequently

The upstream promoter sequences of each *var* gene can be classified into three main ups groups, upsA, upsB and upsC (Rask *et al.*, 2010). Earlier studies on a much smaller dataset (Kraemer *et al.*, 2007), based on sequence similarity, proposed that *var* gene recombination preferentially occurs within the same ups group. Using our method, which to our knowledge is the first systematic attempt to detect recombinants in *var* genes in natural parasite populations, we found considerable evidence supporting this hypothesis. Our results are summarized in Table 1.

**Table 1.** Proportions of recombinations from the same ups groups and DBL*α* subclasses

	Parent–child	Parents	Family
UpsA versus upsB/C	99.7% (92.5%)	98.9% (85.0%)	98.5% (85.0%)
UpsA, B and C	85.3% (75.4%)	65.5% (50.9%)	51.1% (50.9%)
DBL*α*	58.8% (53.9%)	31.0% (7.9%)	20.6% (7.9%)

*Note*: Expected proportions are given in brackets. All *P*-values are highly significant ($<2.2\times 10^{-16}$) except for the entry marked in red (*P *=* *0.2734).

We calculated the proportion of recombination triples which have one parent and the child, both parents and both parents and the child belonging to the same ups group (‘Parent-child’, ‘Parents’ and ‘Family’ in Table 1). With one exception, we found that the parents and/or the child of a recombination were significantly more likely ($p<2.2\times 10^{-16}$ from $\chi^{2}$ tests) to belong to the same ups group, compared with a (conservative) null model where the parents have independent groups, but the child shares the group of one of its parents. (Under a more liberal model where the child group is also independent, all *P*-values are highly significant.) Our results strongly reinforce the conclusions of earlier studies, and provide more precision with the division into three ups groups.

We also considered the proportions of identified recombinants in each ups group. We found that there was a significant difference in the proportions of recombinants in the three groups ($p=2.193\times 10^{-7}$ from a $\chi^{2}$ test), with upsA having the least proportion of recombinants, and upsC the most (82.3%, 84.9% and 87.6% from A, B and C, respectively).

#### 3.2.2 Proportions of recombination differ among domain subclasses

DBL*α* sequences can also be classified according to sequence similarity into 33 subclasses (DBL*α*0.1–24, DBL*α*1.1–8, DBL*α*2). These subclasses are strongly associated with ups groups; however, they also provide greater resolution in dividing the sequences. We thus repeated our earlier analyses with regards to the subclasses. As with ups group, we found a significant (all $p<2.2\times 10^{-16}$) increase in recombinations with one parent and the child, both parents and both parents and the child from the same domain subclass (Table 1).

**Fig. 4.**
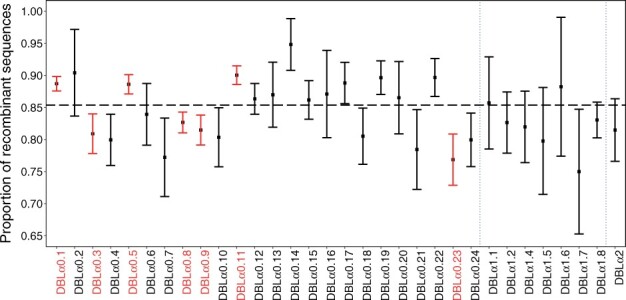
Proportions (and 95% confidence intervals) of recombinants for each DBL*α* subclass. Subclasses which are significantly different from the overall average (under a correction for multiple testing) are highlighted in red. The horizontal dashed line displays the overall proportion of recombinant sequences in the entire dataset

We next considered the proportions of identified recombinants in each subclass (Fig. 4). We identified seven subclasses (DBL*α*0.1, 5 and 11 were too high, while DBL*α*0.3, 8, 9 and 23 were too low) which were significantly different from the average under a Bonferroni correction for multiple testing. Of particular note is the DBL*α*0.1 subclass, which has been noted to involve more recombinations than other subclasses (Claessens *et al.*, 2014). We suggest that these subclasses should be explored further to determine if there are some biological factors that may explain these results.

We also investigated the proportion of recombinants among individual isolates, and among the two broad catchment areas in the Bongo District (Soe and Vea/Gowrie) that the isolates were collected from. We did not detect any significant differences here (see Supplementary Section S3.2).

#### 3.2.3 Non-recombinant DBL*α* types are more conserved than recombinant types

It is well known (Rougeron *et al.*, 2017; Ruybal-Pesántez *et al.*, 2017) that some DBL*α* types are highly conserved (appear in many different isolates) in a population (or even globally, Tonkin-Hill *et al.*, 2021). On the other hand, many other types only appear rarely, or even once. We hypothesize that non-recombinant types are more ‘stable’ than recombinants, and thus may be more highly conserved.

We investigated this hypothesis via the recombinants identified by our method. First, we compared the observed frequencies in the dataset of the recombinants to the non-recombinants; we found that non-recombinants occurred significantly more often (average 4.2 versus 3.7, *P *=* *0.021 from a Wilcoxon rank sum test).

We also considered if there is a difference in the proportions of frequent DBL*α* types in recombinants and non-recombinants. As the frequencies of types are highly right-skewed (see Supplementary Fig. S19), we thresholded the frequencies at various levels to determine if there were particular frequencies where an effect could be noticed. The results are in Table 2. We found that for a threshold frequency of 5, there were significantly fewer frequent recombinants than non-recombinants; however, this effect becomes less noticeable for larger thresholds. This suggests that there is a high proportion of recombinants which appear very few times in the dataset; these are potentially relatively recent recombinants, which may have not been established in the population.

**Table 2.** Proportions of frequent (larger than the threshold) recombinant and non-recombinant DBLα types

Threshold	5	10	15	20
Recombinants	17.5%	4.5%	2.1%	1.3%
Non-recombinants	21.0%	6.0%	2.3%	1.6%
*P*-value ($\chi^{2}$ test)	0.006	0.047	0.666	0.634

#### 3.2.4 Breakpoint positions are associated with homology blocks

It is known that a number of semi-conserved homology blocks (HBs) occur frequently in *var* genes (Rask *et al.*, 2010). These HBs recombine at exceedingly high rates (Freitas-Junior *et al.*, 2000; Taylor *et al.*, 2000), and are known to be useful in predicting disease severity (Rorick *et al.*, 2013). We thus investigated the patterns of recombination in DBL*α* types in relation to these homology blocks.

**Fig. 5.**
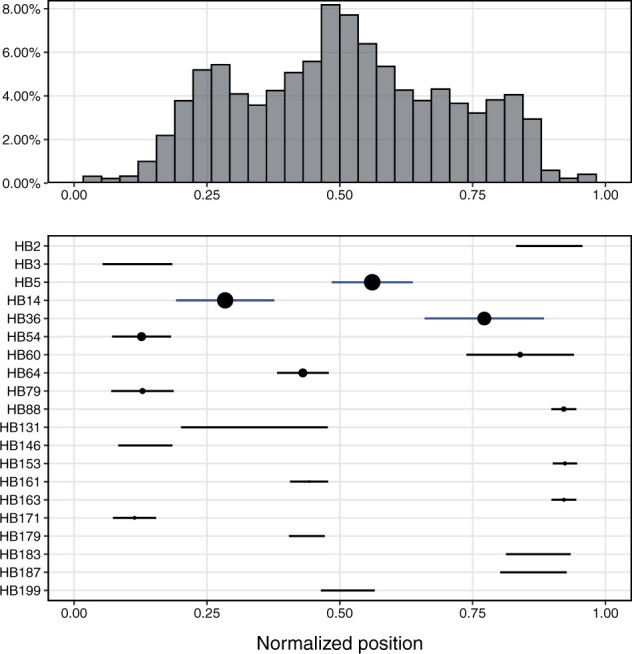
Positions of recombination breakpoints. (Top) The histogram of relative breakpoint positions of recombinations. (Bottom) The position of the most common homology blocks, with circle size proportional to frequency. The three most frequent homology blocks (HB5, 14 and 36) are highlighted in blue

The positions of recombination breakpoints, as found by the JHMM method, are shown in Figure 5. Of particular note is:

- The recombination rate is not constant throughout the sequence, but displays three distinct peaks spaced in roughly equal intervals. These peaks clearly correspond to the three most frequent homology blocks, HB5, 14 and 36, with the height of the peak also corresponding to the frequency of the HB.
- The frequency of breakpoints drops sharply toward either end of the sequence. This is an artifact of the method and does not imply that the recombination rate is lower there; we cannot recognize a recombination which is close to one end of the sequence.

This reinforces the biological theory that recombination occurs within short identical segments (Sander *et al.*, 2014).

## 4 Discussion

In this article, we have developed a statistical method to detect recombinant sequences from a large set of genetic sequences without requiring a multiple alignment or a reference panel. We can also assess the reliability of the inferred recombinants with a bootstrapping-based tool. Simulations show that our method performs very well even when there is a high recombination rate, long sequences or a large dataset. Crucially, it maintains its accuracy in the presence of insertions and deletions, where methods that require an alignment would normally fail. In a study of DBL*α* domains of *var* genes, comparisons between recombinant and non-recombinant DBL*α* types reveal a series of biologically meaningful results; we find evidence for the hypothesis that recombination is more frequent within ups groups, but also find that it is more frequent within domain subclasses. We also find novel results that recombinants differ from non-recombinants both in their representation in domain subclasses, and in their levels of conservation.

While our method is not strictly an alignment-free tool, it carries several advantages over methods based on a full multiple sequence alignment. Our method mostly aligns segments which are closely related to each other, thus increasing the reliability of the alignments; as datasets increase in size and variability, it will become more difficult to construct a reliable full alignment for all sequences. Moreover, our method only attempts to align three sequences at once, again saving time and increasing reliability. By identifying recombination triples directly from the JHMM, our method also avoids having to examine all possible triples of sequences one by one.

As noted above, our method is designed to only detect recent recombinants, which have not yet diverged in the dataset. For example, if a more ancient recombination produces a lineage that diverges into two sequences, they will be preferentially matched to each other by the JHMM, and it is possible that no recombination will be detected. The initial clustering of DBL*α* tags into types at 96% similarity (a standard part of the preprocessing pipeline) may help in this regard, as the lineages must diverge beyond this threshold to be distinguished. The use of different clustering thresholds may affect the results, potentially unlocking access to signals of older recombinations.

Note that it is uncertain how long a recombinant will remain recent for, and this may well depend on sampling coverage and sample size. For example, although recombination events have been reported on timescales of several years (Claessens *et al.*, 2014), a recombinant may continue to be ‘recent’ for far longer than that. The Ghana dataset studied in this article is the first of a longitudinal dataset collected over several seasons, which may give insight into the frequency and patterns of recombination on epidemiological timescales; this is the subject of current work.

Furthermore, there is an implicit assumption that recombinations do not ‘interact’ with each other, i.e. that they are sufficiently far apart either in the evolutionary network or in the genome that we can decompose the dataset into recombinant triples and assess those independently. This is a strong (and perhaps unrealistic, in the context of genes which have a high recombination rate) assumption which we make to obtain a tractable algorithm. As seen from our results, we do appear to obtain good accuracy with our detections even in cases where this assumption might not hold; assessing the exact impact of this assumption on our results is also the subject of future work.

Although our methods are motivated primarily by the highly recombinant *var* genes, our approach is not restricted to these genes, but could be used for any genes which are recombinant but lack a reliable alignment or reference panel. The scalability of our method means that it will be applicable even to large datasets, thus holding great promise for broader applications.

## Supplementary Material

btac012_Supplementary_Materials
