# Supplementary material for: An accurate method for identifying recent recombinants from unaligned sequences
Source: Bioinformatics. 2022 Jan 13;38(7):1823–9. doi: 10.1093/bioinformatics/btac012 (PMC8963311; doi:10.1093/bioinformatics/btac012)
Supplement: btac012_Supplementary_Materials [file btac012_supplementary_materials.pdf]

An accurate method for identifying recent recombinants from unaligned sequences

Supplementary materials

Qian Feng, Kathryn E. Tiedje, Shazia Ruybal-Pesántez, Gerry Tonkin-Hill, Michael F. Duffy, Karen P. Day, Heejung Shim, Yao-ban Chan

1 Methods

1.1 Identifiability: a phylogenetic perspective

An important component in our method is the ability to identify which member of a triple is the true recombinant. It is important to note that the JHMM method does *not* identify the recombinant, but instead finds the (segments of) extant sequences which are the most closely related to the target sequence.

This can be illuminated by considering an explicit phylogenetic network (Huson *et al.*, 2010) with three aligned sequences and one recombination as an example, as shown in Figure S1. Here, we can translate a phylogenetic network to the corresponding mosaic representations, assuming the JHMM method estimates the distances between sequences perfectly. It can be seen that the same mosaic structure can result from networks with different recombinants.

In fact, as discussed at length by Pardi and Scornavacca (2015), this is an unavoidable problem with the identifiability of phylogenetic networks; networks cannot be distinguished solely by the topologies of displayed trees, which the output of the JHMM method is dependent on. The solution, as given in that paper, is to use (inferred) branch lengths to distinguish between the networks, and thereby identify the recombinant.

When the phylogenetic network only consists of three sequences and one recombination (as in Figure S1), it is easy to translate the network to the JHMM output, and thus use it to find the recombinant. However, the problem rapidly becomes much more complicated with more sequences and/or recombinations, and indeed for ancestral recombinations (predating a divergence) it's not even clear how to define an extant 'true recombinant'. To avoid this problem, we only identify triples of sequences, and assume that only one recombination occurs in the recent evolutionary history of each triple. For large datasets, we are essentially assuming that recombinations are 'sufficiently far apart' either in the network or in the genome that they do not interact with each other.

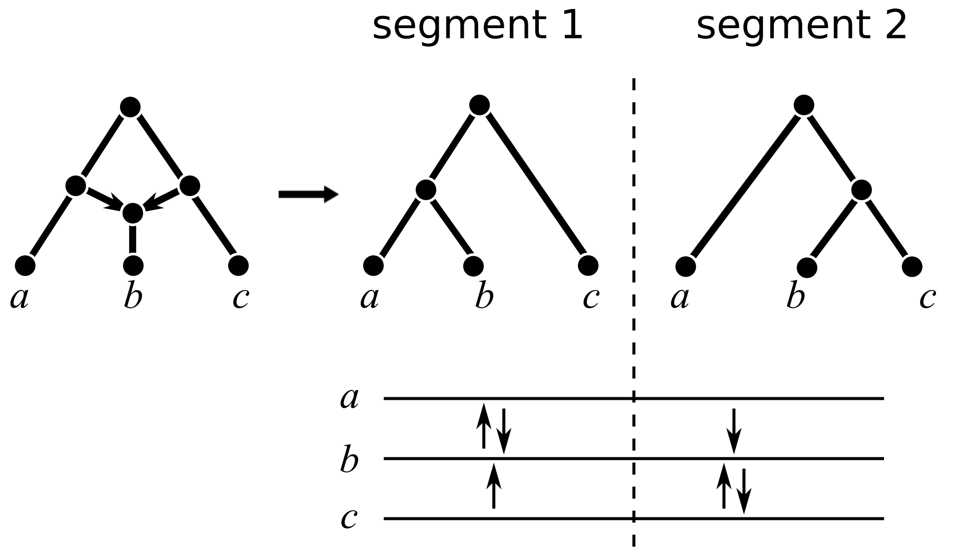

(a) Sequence b is the recombinant

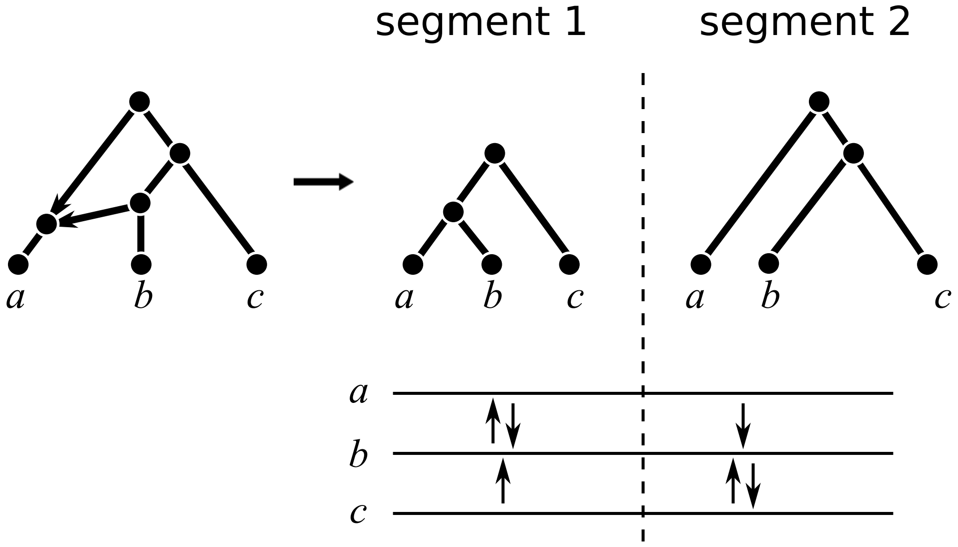

(b) Sequence a is the recombinant

Figure S1: **Identifiability of networks from the JHMM output.** Here, two networks with different recombinants produce the same profile tree topologies, and thus the same JHMM output. The JHMM output is depicted below the profile trees, with arrows from each target segment pointing to the matching source segment (so, for example, if b is the target sequence, it is matched to source sequence a in segment 1 and c in segment 2 in both cases). Both cases produce identical JHMM output: in particular, sequence b is matched to two different source sequences even though it is not necessarily the recombinant.

From a phylogenetic perspective, we can see that when this assumption holds, identifying only triples breaks down a complicated network into repeated cases of a three sequence–one

recombination network, for which we can identify the recombinant. See Figure S2 for an example of this.

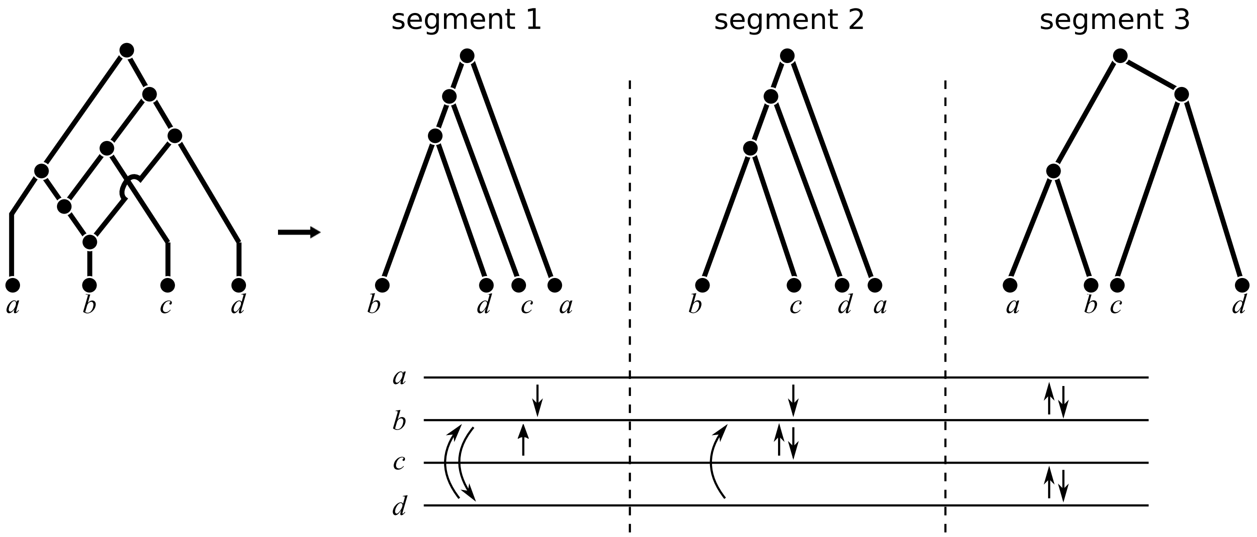

Figure S2: **Decomposing a network into triples.** At the first breakpoint, the triple $\{b, c, d\}$ is identified from target sequence b, while at the second breakpoint, $\{a, b, c\}$ is identified from sequence b, and $\{b, c, d\}$ from sequences c and d. In all cases, distance-based recombinant identification will obtain the correct recombinant (b at both breakpoints).

2 Simulation results

2.1 Effect of parameters

The parameters that we vary in the simulations, and their ranges, are shown in Tables 1 and 2. We vary one parameter at a time, holding the remainder at default values (shown in bold in the tables). We now consider the effect of each parameter in turn.

Recombinant proportion As the proportion of recombinants increases, sensitivity is stable at around 80%, while specificity decreases (Figure S3). Here, more recombinant sequences result (correctly) in a higher number of recombinations detected. It appears that the proportion of true recombinants extracted from the recombinant triples remains largely the same (constant sensitivity); however, there are proportionally more false detections as the number of non-recombinants decreases, resulting in a lower specificity.

Number of recombinations per recombinant As shown in Figure S4, the datasets where there are more recombinations per recombinant sequence appear to have a higher sensitivity, and slightly lower specificity. As with recombinant proportion, an increase in the number of recombinations results (correctly) in more inferred recombinations; unlike

Table 1: **General simulation parameters (no indels).** We vary each parameter in turn while holding the others fixed at the default values (in bold).

**Parameter**	**Values**
① Proportion of recombinant sequences (%)	10, 20, 30, 40, **50**, 60, 70, 80, 90
② Average number of recombinations per recombinant sequence	1.0, 1.1, 1.2, 1.3, 1.4, **1.5**, 1.6, 1.7, 1.8, 1.9, 2.0
③ Dataset size (sequences)	100, 150, **200**, 250, 300, 350, 400, 450, 500
④ Sequence length (AA)	100, 150, **200**, 250, 300, 350, 400, 450, 500
⑤ Mutation rate (substitutions/site/coalescent unit)	0.1, 0.2, 0.3, 0.4, **0.5**, 0.6, 0.7, 0.8, 0.9, 1.0
⑥ Amino acid evolution model	AB (Mirsky *et al.*, 2015), DAYHOFF (Dayhoff *et al.*, 1978), JTT (Jones *et al.*, 1994), LG (Le and Gascuel, 2008), MTMAM (Yang *et al.*, 1998), **WAG** (Kingman, 1982)

Table 2: **Indel simulation parameters (default values in bold).** Insertions and deletions are simulated at the same rate, with lengths according to a negative binomial distribution with variance 10.

**Parameter**	**Values**
⑦ Indel rate (expected number of indels/substitution)	0.1, 0.2, **0.3**, 0.4, 0.5
⑧ Mean indel size (AA)	3.7, 5.2, **6.0**, 6.6, 7.0

that case, the number of true recombinants remains the same here. It appears that the 'extra' detections are mostly correct, which results in a greater proportion of true positives (sensitivity increases) and a relatively stable specificity.

We also conducted a further analysis by matching the distribution of the number of recombinations per recombinant to the Ghana dataset (see Supplementary Section 3.4 and Figure S22 for more details). Our results indicate that, despite a low specificity (40.0%), a high sensitivity (89.0%) still demonstrates the applicability of our algorithm to real data.

Dataset size Dataset size does not appear to have a drastic effect on the sensitivity of the method, while specificity increases slightly (see Figure S5). It is to be expected that performance increases slightly as information accumulates across a larger dataset, but it is unclear why this is only expressed in the specificity here.

Sequence length Datasets with longer sequence length have much higher sensitivity, and slightly lower specificity (Figure S6). It seems (Figure S7) that as sequence length increases, the number of recombinations detected also increases, even though the true number of recombinations remains the same. This increase in detections, combined with a fixed percentage of recombinants, results in a effect similar to that seen for the number of recombinations per

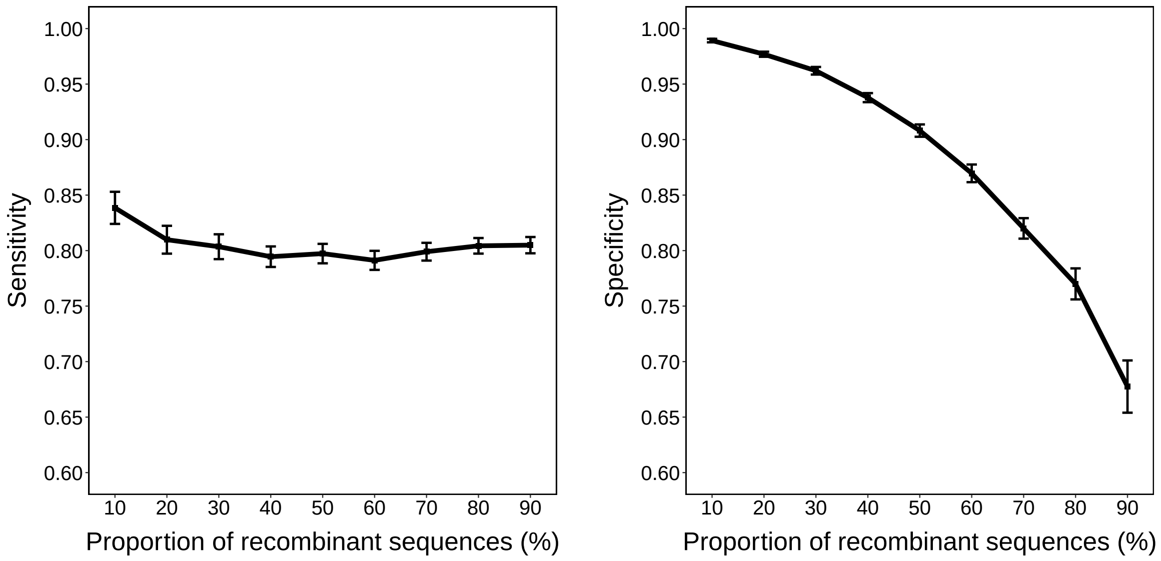

(a) (b)

Figure S3: **Mean sensitivity and specificity (with 95% confidence intervals) for varying proportions of recombinant sequences.**

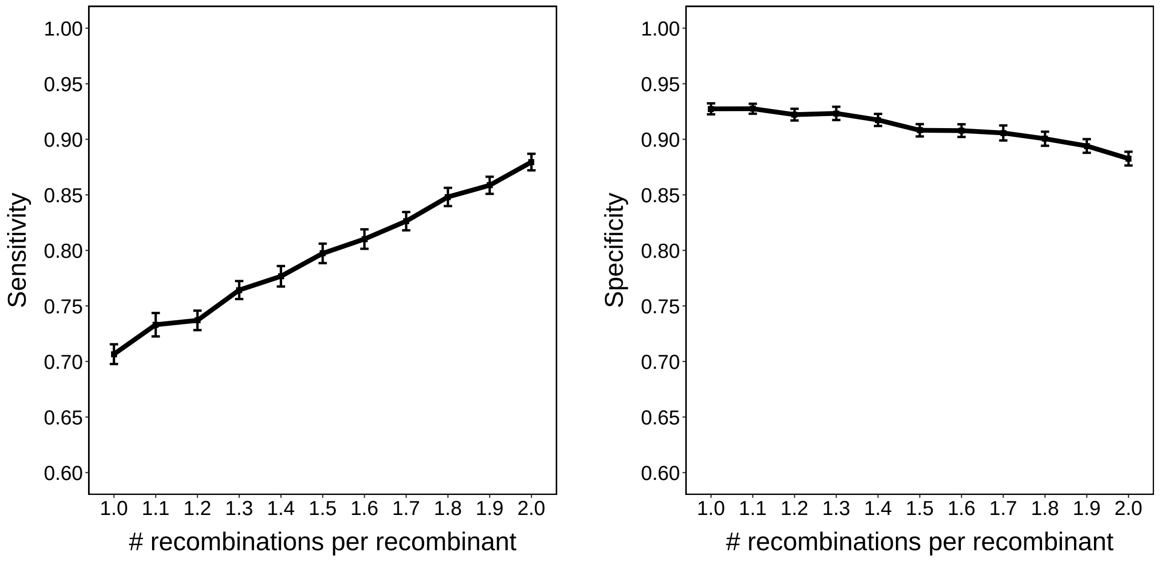

(a) (b)

Figure S4: **Mean sensitivity and specificity (with 95% confidence intervals) for varying numbers of recombinations per recombinant sequence.**

recombinant: an increase in sensitivity and a slightly decreasing specificity.

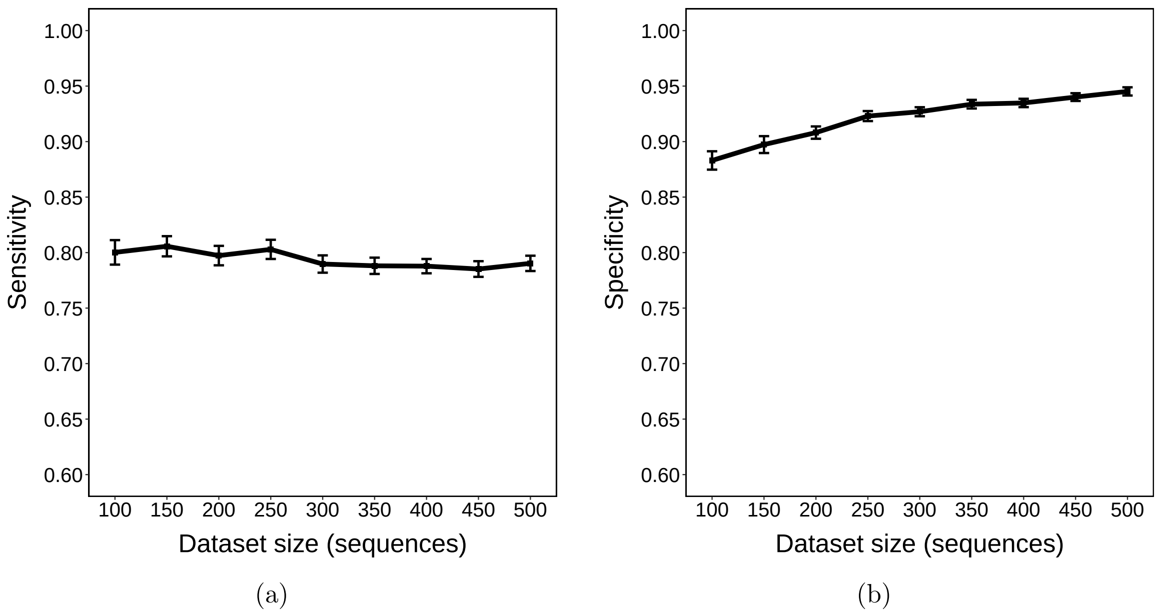

Figure S5: **Mean sensitivity and specificity (with 95% confidence intervals) for varying dataset size.**

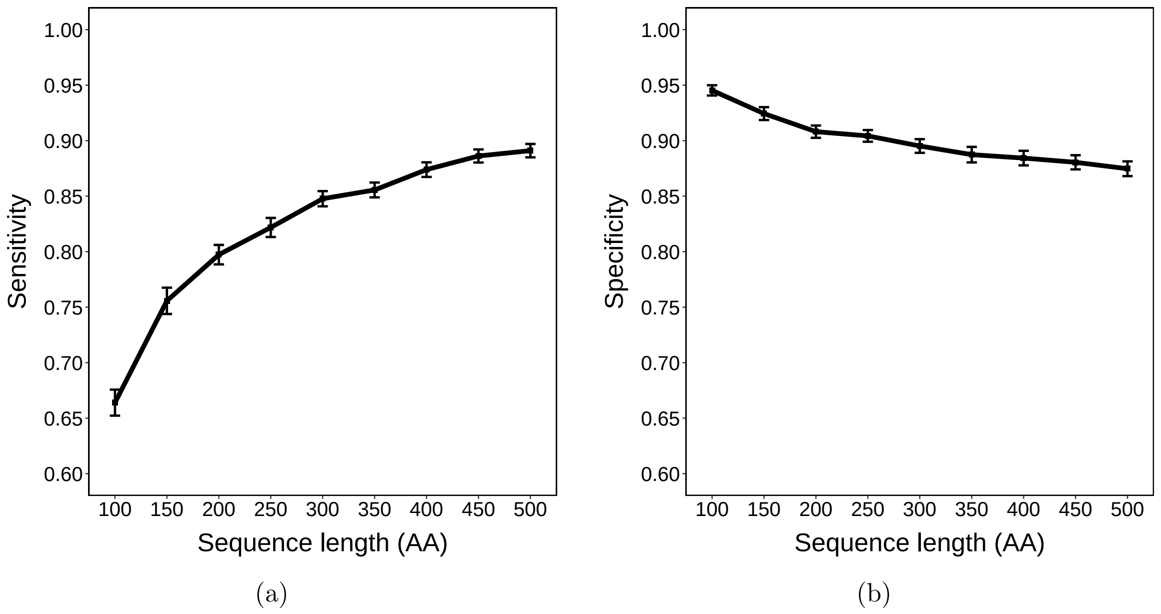

Figure S6: **Mean sensitivity and specificity (with 95% confidence intervals) for varying sequence length.**

Mutation rate As the mutation rate increases, the sensitivity of the method rapidly increases before levelling out (Figure S8). This makes sense, as if the number of substitutions

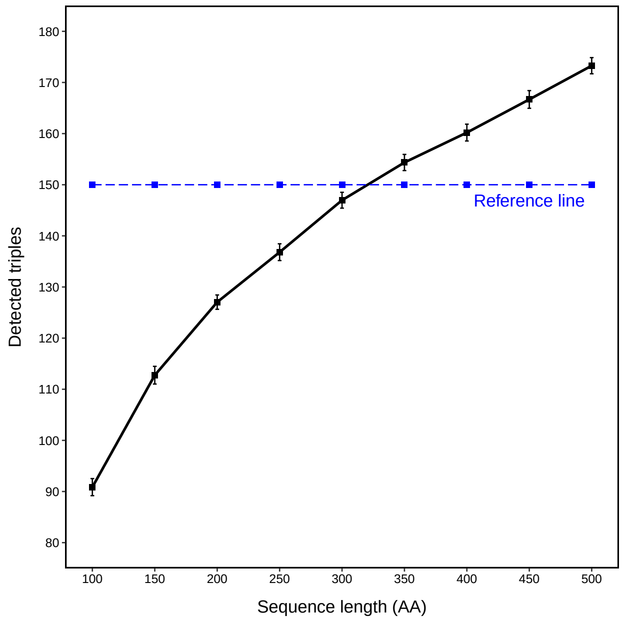

Figure S7: **The number of recombinant triples detected by our algorithm for varying sequence length.** The reference line indicates the true number of recombinant triples in the dataset.

is too low, the sequences are difficult to distinguish from each other, which makes the results from the JHMM unreliable. Conversely, as the number of substitutions grows, it also becomes more difficult to identify sequences which are closely related to each other, resulting in a decrease in specificity.

Evolution model The method appears to be robust to the stochastic model of amino acid evolution (Figure S9).

Indel size When indels are included in the generating process, accuracy is not affected by the size of the indels (Figure S10).

Running time As expected, the only parameters which affect the running time of the algorithm are dataset size and sequence length. In Figure S11, we show the running time of the simulations for each replicate (without parallelisation; see below). The running time appears to grow quadratically with respect to both dataset size and sequence length (the slopes of regressions on the log-log data are 2.09 and 2.28 respectively). This compares favourably to many recombinant detection methods which are based on examining all triples of sequences, and are thus $O(n^3)$ in the dataset size.

While the total running time becomes quite large at even moderate dataset sizes, the algorithm is easily parallelisable in a relatively naive way. The main computational task of the algorithm is in the determination of Viterbi paths for every sequence in the dataset with

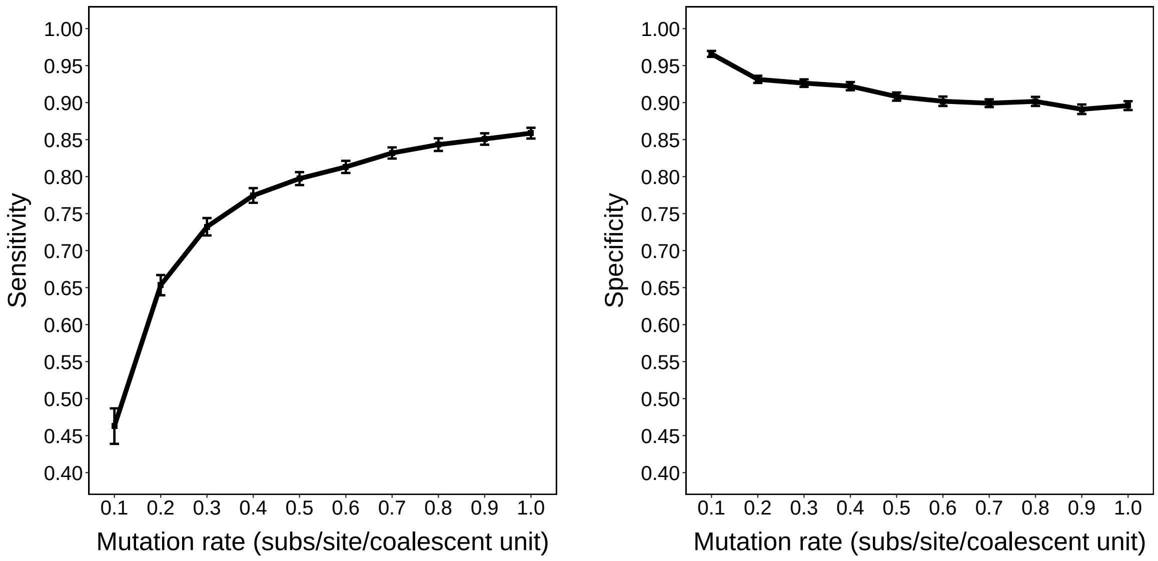

(a) (b)

Figure S8: **Mean sensitivity and specificity (with 95% confidence intervals) for varying mutation rate.**

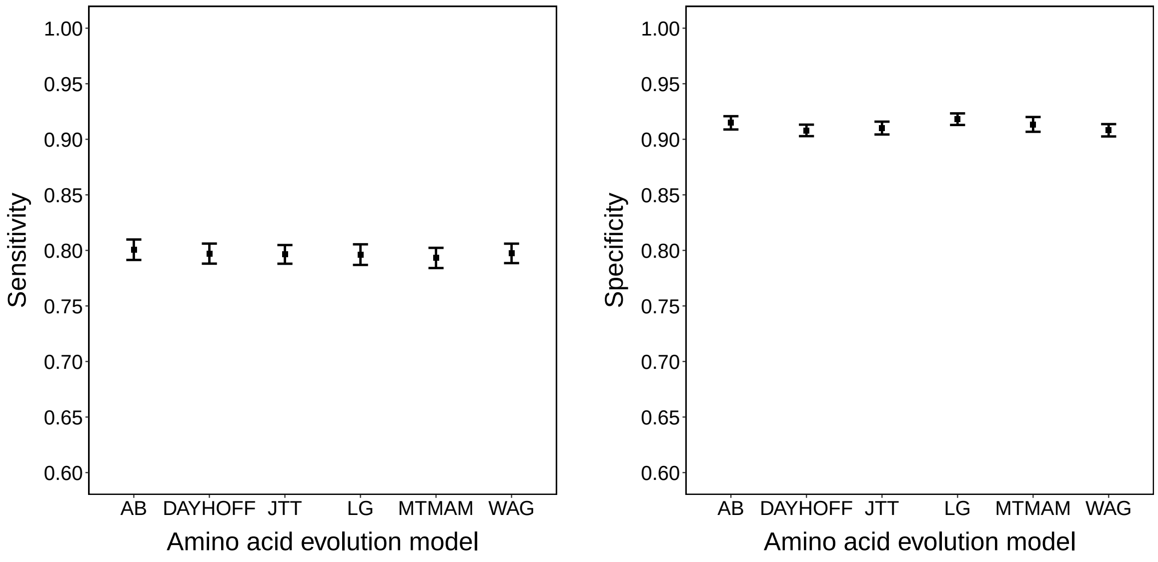

(a) (b)

Figure S9: **Mean sensitivity/specificity (with 95% confidence intervals) for each model of amino acid evolution.**

respect to all other sequences. This is used for both training the JHMM, and calculating its final output. By computing the Viterbi paths for each sequence in parallel, we can achieve

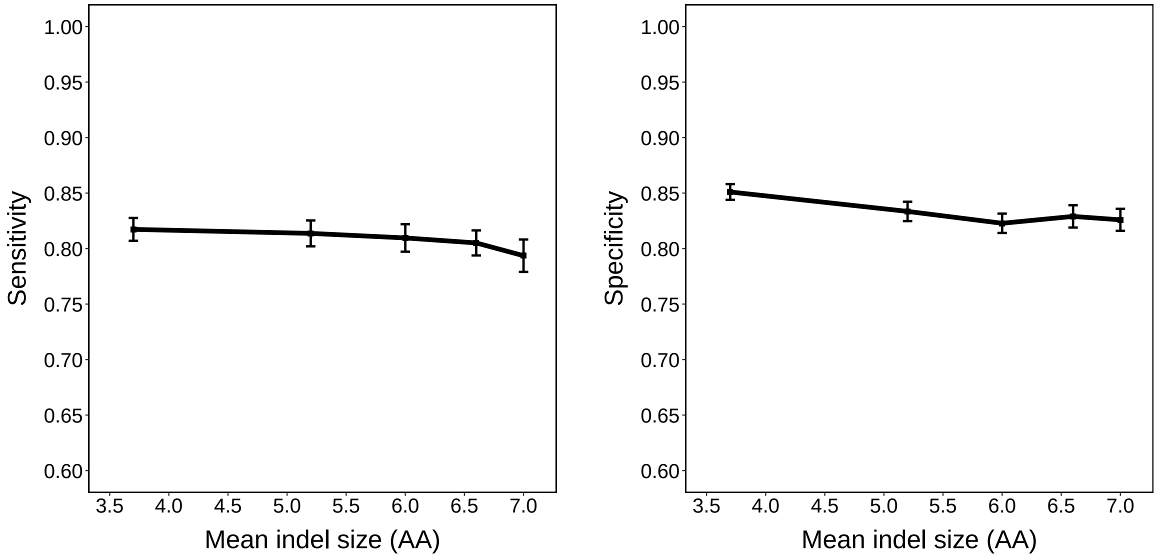

(a) (b)

Figure S10: **Mean sensitivity and specificity (with 95% confidence intervals) for varying indel size.**

massive savings in real time; for example, the *var* gene dataset can be analysed in a tractable amount of time even with many more sequences.

On the other hand, this parallelisation does not produce any benefits as the length of the sequences grow longer. Thus our algorithm is more suited to the analyses of massive datasets of relatively short sequences.

2.2 Comparison with other methods

We compared our method with the recombinant detection methods 3SEQ (Boni *et al.*, 2007; Lam *et al.*, 2018), Chimaera (Posada and Crandall, 2001), GeneConv (Padidam *et al.*, 1999), MaxChi (Smith, 1992), RDP (Martin and Rybicki, 2000), and Siscan (Gibbs *et al.*, 2000). All but the first method are implemented in RDP5 Beta 6 (Martin *et al.*, 2020).

As these methods mostly accept aligned DNA sequences as input, we simulated DNA sequences with length 200nt under the F81 substitution model (Felsenstein, 1981). Other parameters followed the default simulation settings in Tables 1 and 2. We simulated both with and without indel events, then aligned the resulting sequences with MUSCLE v3.8.31 (Edgar, 2004) for methods requiring an alignment.

As our method does not utilise p-values, in order to ensure a fair comparison we thresholded the p-values output by other methods so that the specificity (false detection rate) of all the methods are the same. We then compared the sensitivities of each method.

In addition, we also compared both the sensitivity and specificity of all the methods for their default settings (Figure S12). With indels simulated, our method is clearly superior

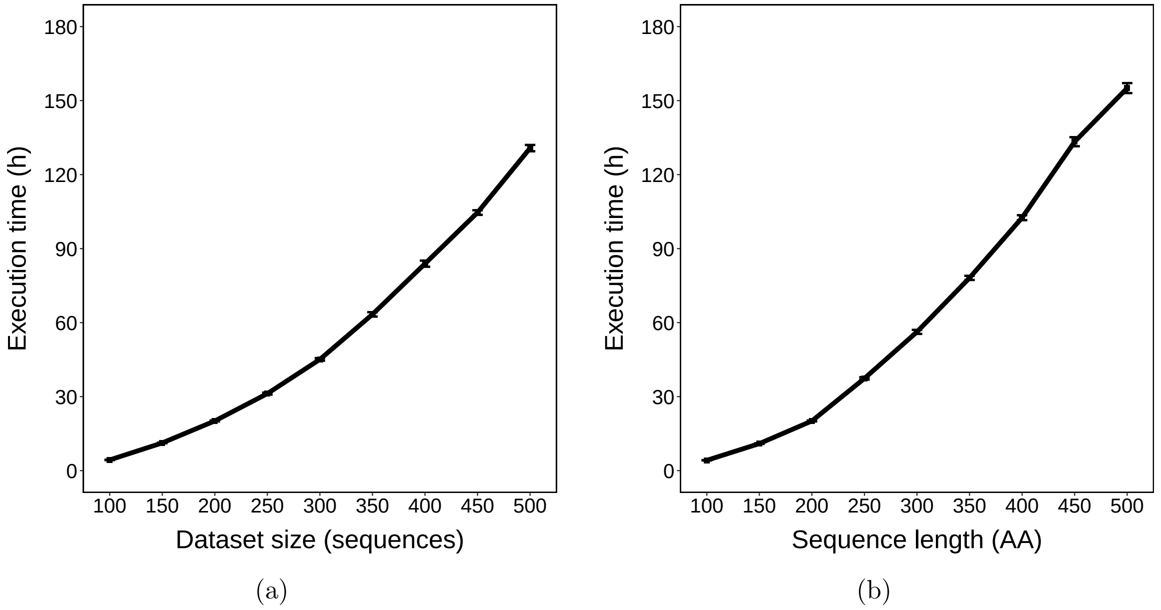

Figure S11: **Average running time per replicate (and 95% CIs) for varying dataset size (left) and sequence length (right).**

in both sensitivity and specificity, as expected; with no indels simulated, methods based on aligned sequences perform better than before, but our method is still superior.

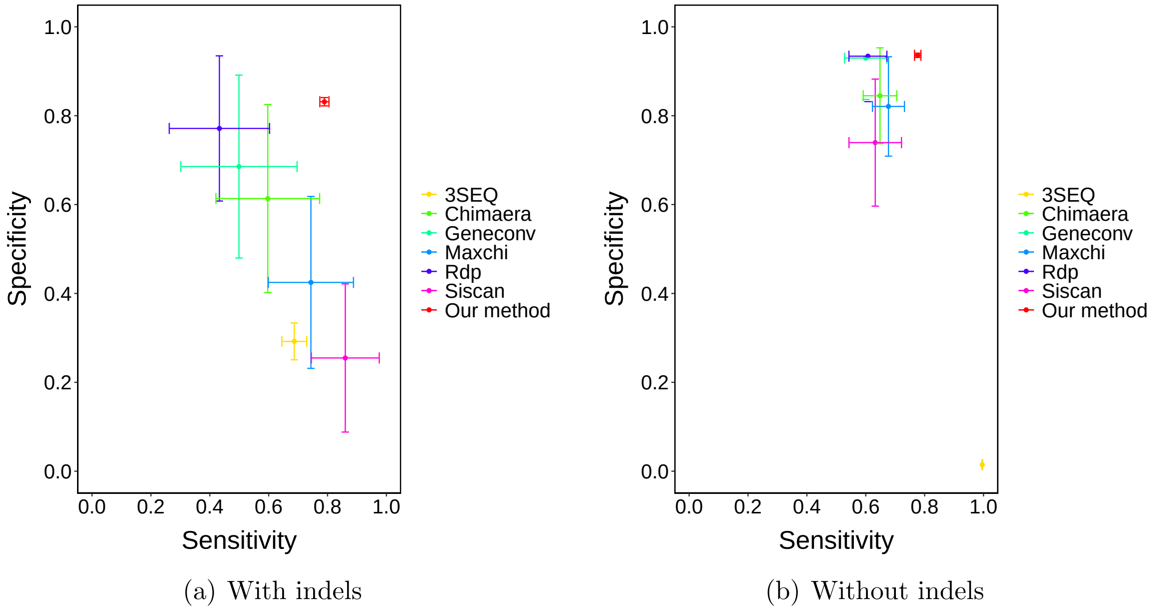

Figure S12: **Sensitivity and specificity (and 95% CIs) under default parameters with and without indel events.**

2.3 Ancient recombinations

Our simulations are designed to only contain 'recent' recombinations, that is recombinations which only descend to one sequence in the dataset. This allows us to have complete control over the proportion and make-up of recombinants, and to unambiguously distinguish between recombinants and non-recombinants. On the other hand, it is possible that our method may be hindered by the presence of ancient recombinations which descend to a number of sequences in the dataset.

To test this, we used the `msprime` software to simulate sequences under the full coalescent with recombination; this allows recombinations to occur throughout the evolutionary history of the sequences. We use default values for the simulation parameters (apart from the proportion of recombinant sequences and average number of recombinations per recombinant), and vary the recombination rate, producing varying proportions of recombinant sequences in the dataset. We then determined each sequence as a recent recombinant if and only if it is the only extant descendant of an ancestral segment produced by a recombination (i.e., a segment surrounding the recombination breakpoint).

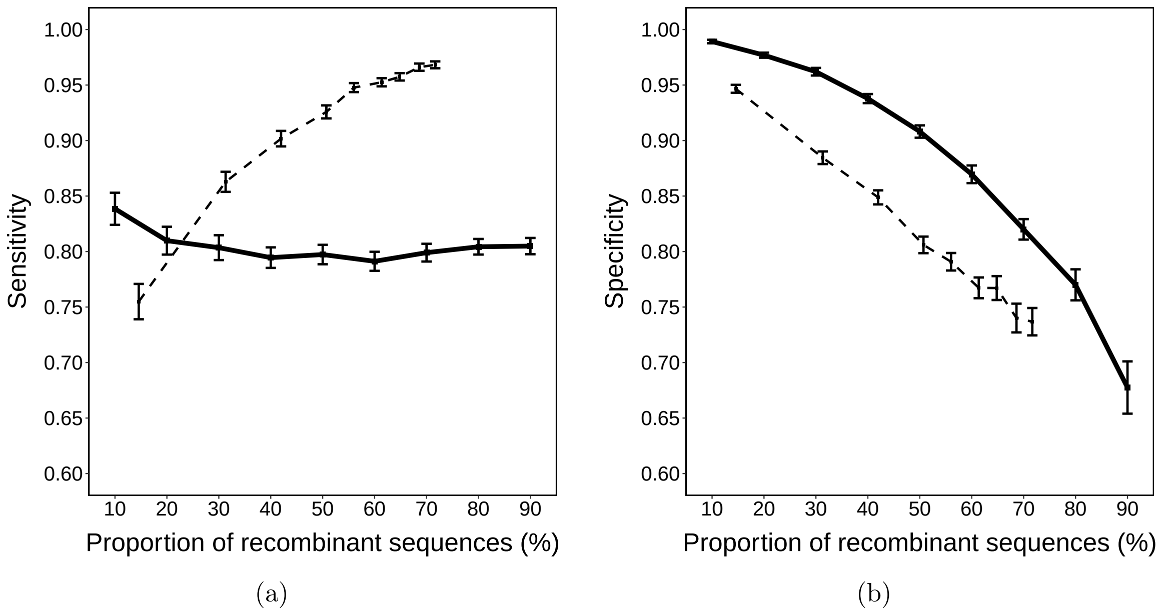

Figure S13: **Sensitivity and specificity (and 95% CIs) for recent recombinations only (solid lines) and recombinations allowed throughout (dashed lines), for varying recombination rate.**

We observe that our method still retains a lot of power to detect recent recombinants under this scenario, with slightly higher sensitivity and slightly lower specificity compared to our previous simulations. Indeed, the sensitivity improves with the recombination rate; it appears, rather pleasingly, that our method has some ability to even detect the signal of older recombinations.

2.4 Support values

In addition to detecting recombinants, we also calculate support values for each detection using bootstrapping. Here, we verify that the calculated values are indeed effective for this purpose. For our simulations, we calculate the support values for each of the correct detections, as well as each of the false positives. The distributions of the support values for the default parameters are shown in Figure S14. Here, we can see that there is a clear separation between the distributions of support values for the true and false positives; while the values for both are relatively high, the support values for true detections are overall much higher. Similar patterns are seen among all the remaining parameter settings (Figures S23–S30).

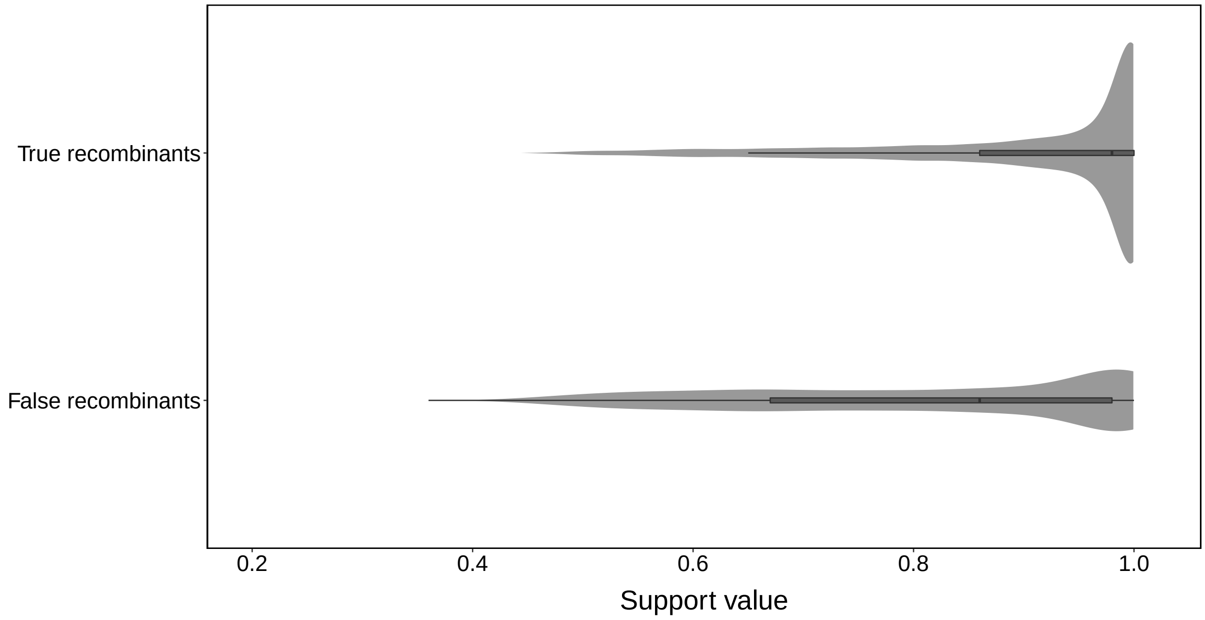

Figure S14: **Distributions of support values under default parameters without indel events.**

This suggests that we can use a threshold on the support value to refine our detections. This is reasonable if we wish to reduce false positives; however, in practice we found that applying a threshold also reduced true positives (as expected) to an extent which lowered the overall accuracy of the method, so we have elected not to apply it here. Instead, we suggest that the support value be used to assess the confidence which should be placed in individual recombinant detections of interest.

2.5 Accuracy of the JHMM method

The JHMM method of Zilversmit *et al.* (2013) forms a key part of our method to detect recombinants. Until now, there has not been a systematic study of the accuracy of this method. Two key outputs of this method are the locations of the inferred recombination

breakpoints, and the estimated recombination parameter ρ. Here, we study the accuracy of these inferences for our simulated datasets.

Recombination breakpoints For each recombination, we calculate the distance between the true and inferred breakpoints. For ease of comparison, we restrict this analysis to the case where each recombinant sequence has exactly two parents (one recombination), which avoids the problems of matching breakpoints in the same sequence to each other.

We find in general (see Figure S15) that the breakpoints are very accurately inferred, with 38.4% of all breakpoints inferred exactly, and 75.0% being at most 5AA from the true value. There is also a slight but noticeable positive bias, where the inferred breakpoints tend to be slightly to the right of the true breakpoints (Figure S16). This can be best explained by noting that the JHMM method infers the best (Viterbi) path from left to right, and recombinations are considered relatively unlikely; hence a recombination will tend to be inferred slightly later than it actually is, particularly if both parents' sequences are identical around the breakpoint.

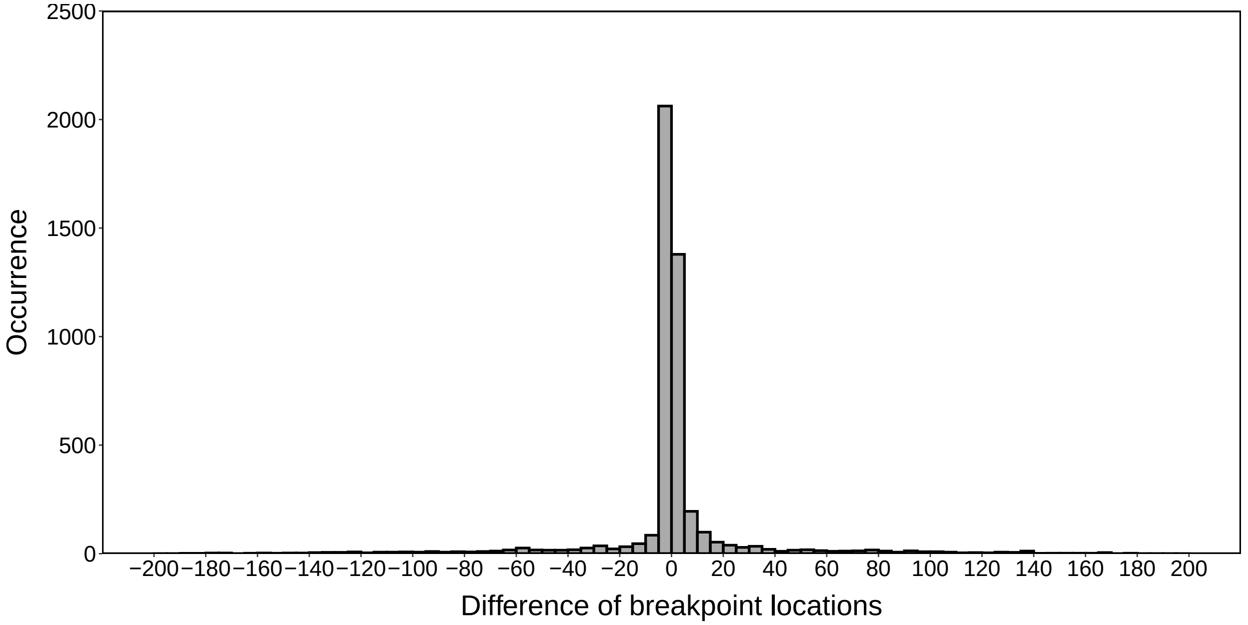

Figure S15: **Breakpoint inference error of the JHMM method under default simulation parameters.**

Finally, we note that the breakpoint accuracy appears to be very robust to indel events; this is expected, since the method explicitly accounts for these events.

Recombination rate The parameter ρ, the probability of switching between source sequences after any character, is directly related to the recombination rate in the dataset (although it does not provide a rate in terms of time dimension). As such, an accurate estimate of ρ is valuable for molecular phylogeneticists. We observe in our simulated datasets

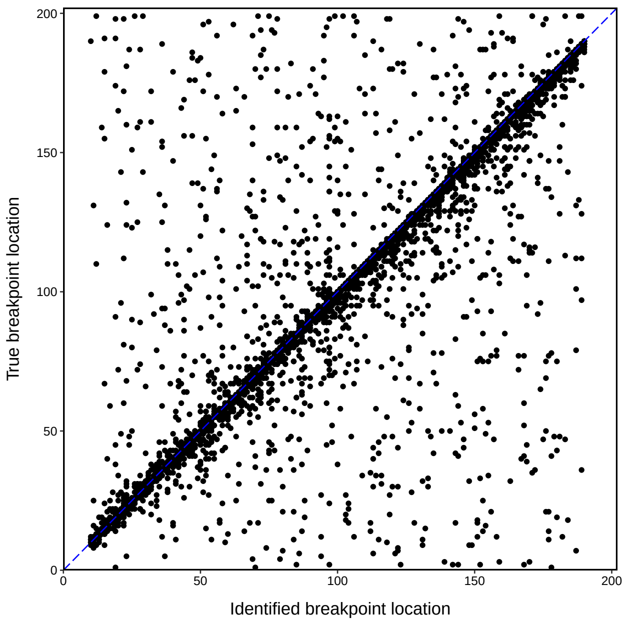

Figure S16: **Breakpoint inference of the JHMM method under default simulation parameters.** Most points cluster around the line $y = x$, indicating a high accuracy of breakpoint inference. However, there is a slight positive bias in the identified breakpoint location, particularly for breakpoints which occur later in the sequence.

(Figures S31-S34) that the inferred values of ρ provide an accurate estimate of the recombinaton rate.

On the other hand, the inferred ρ can also be affected by mutation rate (Figure S17) and (to a lesser extent) indel events (Figures S35-S36); here, an increasing rate of indel events leads to some of them being mistaken for recombination, distorting the inference of the recombination rate. This indicates that the use of the JHMM to infer the true recombination rate has the potential to be inaccurate.

3 Analysis of DBLα sequences from a cross-sectional study in Ghana

3.1 Data handling

Details on the study population, data collection procedures, and epidemiology have been published elsewhere (Ruybal-Pesántez *et al.*, 2017a; Tiedje *et al.*, 2017; Rorick *et al.*, 2018).

Preprocessing We follow the standard pipeline used in (Ruybal-Pesántez *et al.*, 2017b; Tonkin-Hill *et al.*, 2021). The DNA sequences were first translated into protein sequences, and removed if the resulting sequence contained a stop codon. The protein sequences were

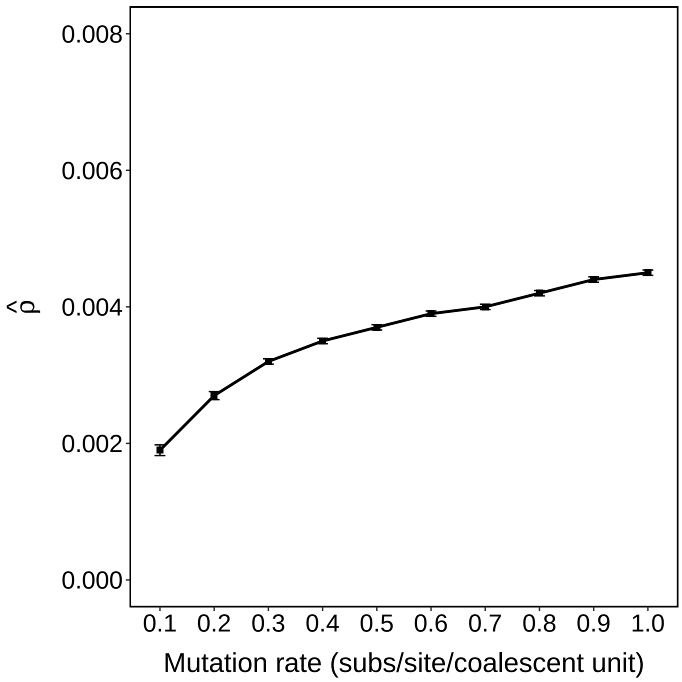

Figure S17: **Estimated ρ (and 95% CI) with varying mutation rate (but constant number of recombinations).**

then clustered with the Usearch software (v8.1.1861) (Edgar, 2010) with a 96% sequence similarity cutoff (Barry *et al.*, 2007). The cluster centroids were then taken as a representative sequence for the clusters, which are known as DBLα *types*. This results in a dataset of 17,335 types, each of which may appear in several isolates.

Identifying recombinants We applied our method to this dataset to detect recombinant types. We detected 14,801 (85.4%) of the DBLα types to be recombinant.

The analysis was run on a high performance cluster at the University of Melbourne (72 Intel(R) Xeon(R) Gold 6254 CPU cores @ 3.10GHz, 768GB RAM). The computation of Viterbi paths for each sequence, which is necessary for both estimating parameters and identifying recombinants, can be performed in parallel; we computed Viterbi paths for 30 sequences (against all other sequences in the dataset) at a time on one core (578 subsets total). The total time taken was 943 minutes; this is broken down in Table 3. By far the largest bottleneck is the computation of the mosaic representations of the sequences (both parameter estimation and computation of the Viterbi paths); once this was completed, the remaining steps are very efficient even for a dataset of this size.

Table 3: **Time and memory consumption of the algorithm on the Ghana dataset.**

	**JHMM parameter estimation**	**Viterbi paths**	**Recombinant identification**
Time (minutes)	644.8	294.9	2.7
Memory (GB)	21.3	21.2	0.1

3.2 Recombinant proportions across isolates and catchment areas

We investigated the proportion of recombinants among individual isolates to determine if there were certain isolates with an elevated or reduced proportion of recombinants. Excluding isolates with less than 20 DBLα types (Ruybal-Pesántez *et al.*, 2017b; Tonkin-Hill *et al.*, 2021) resulted in a total of 158 isolates with a mean of 217.1 DBLα types per isolate (range 33–833). We tested if the average proportion of DBLα types in each isolate was equal to the overall dataset proportion with a t-test with a Bonferroni correction for multiple testing. There were no isolates which had a significantly different proportion of recombinants under this test (see Figure S20).

In addition, 133 isolates (82.6%) were from two catchment areas: Soe and Vea/Gowrie. A χ^2 test showed no significant difference between the proportion of recombinants from these two areas ($p = 0.992$).

3.3 Detection of HBs in recombinant and non-recombinant DBLα types

The location of homology blocks (HBs) in each sequence was obtained using the VarDom server (Rask *et al.*, 2010) with the default cut-off of 9.97 as a threshold to define a match. For each HB, we averaged the leftmost and rightmost relative positions of each occurrence in a sequence to obtain the overall location of the HB.

We identified in total 41 different HBs in the database (mean 5.5, range 1–10 HBs per sequence). HBs are numbered based on the frequency of occurrence (Rask *et al.*, 2010), with HB1 the most frequent. We found that the frequency of HBs in our dataset also decreased with the numbering, with the exception of HB2 and HB3; these HBs are frequent, but lie partially outside the DBLα tag boundaries, making it difficult to positively identify them in the dataset. The most frequent HBs in our dataset were HB5, HB14, and HB36.

To compare sequences directly based on HBs, we used the pairwise HB similarity (Rorick *et al.*, 2013). This is defined as the number of HBs shared between any two sequences, divided by the average number of HBs within a sequence.

We discovered that the number of HBs in recombinant sequences were significantly higher than in non-recombinant sequences (5.5 vs. 5.3, $p < 2.2 \times 10^{-16}$ from Wilcoxon rank sum test). Furthermore, the proportion of sequences containing "important" HBs (5, 14, and 36) were also significantly different between the two groups (83.9% vs. 78.5%, $p = 1.859 \times 10^{-11}$ from χ^2 test), indicating that recombinants tend to have more conserved building blocks. Finally, we found that recombinant sequences had higher pairwise HB similarities with each other than non-recombinants (0.629 vs. 0.618, $p < 2.2 \times 10^{-16}$ from Wilcoxon rank sum test).

3.4 Matching recombination numbers to real data

We performed an additional simulation to match the distribution of the number of recombinations per recombinant sequence to the Ghana data. To do this, we applied the JHMM method to the Ghana data, and extracted the number of source segments matched to each target sequence (Figure S22). From this Figure, we observe that it is extremely rare to have a sequence match to 8 or more source segments (i.e., 7 recombinations), so we do not allow this to happen in our simulations.

The primary difficulty here is that the JHMM method appears to slightly overestimate the number of recombinations, which means that if we simulate recombinations in exactly the same proportion as found from the Ghana data, the JHMM method produces a recombination frequency which is slightly too high. To accommodate this, we tested five sets of probabilities in simulation, and selected the probabilities of (0.02, 0.30, 0.21, 0.23, 0.14, 0.11, 0.00) for 1–7 source segments (0–6 recombinations) for each sequence. This produced a distribution of numbers of recombinations which was similar to the Ghana data.

4 Figures and tables

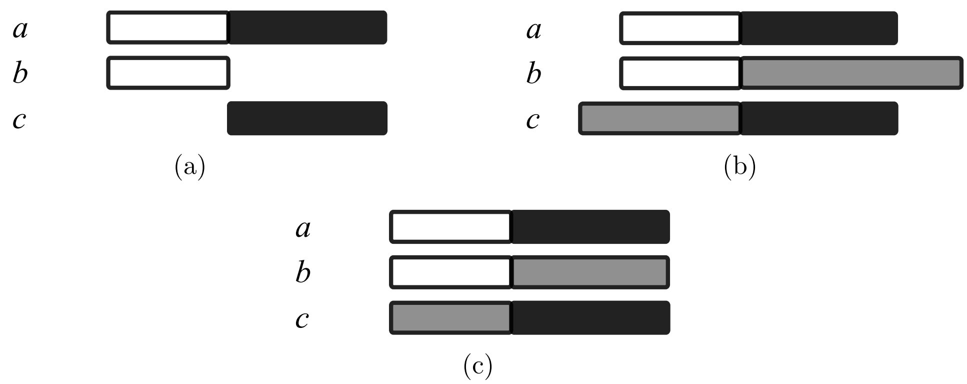

Figure S18: **An overview of calculating a multiple sequence alignment with MAFFT.** (a): A segmental pairwise alignment generated by the JHMM method. Segments from sequence a are aligned to segments from sequences b and c respectively. (b): Using MAFFT, we include the corresponding segment from the third sequence into the pairwise alignment on either side of the breakpoint. (c): By trimming the alignments, we generate a multiple alignment.

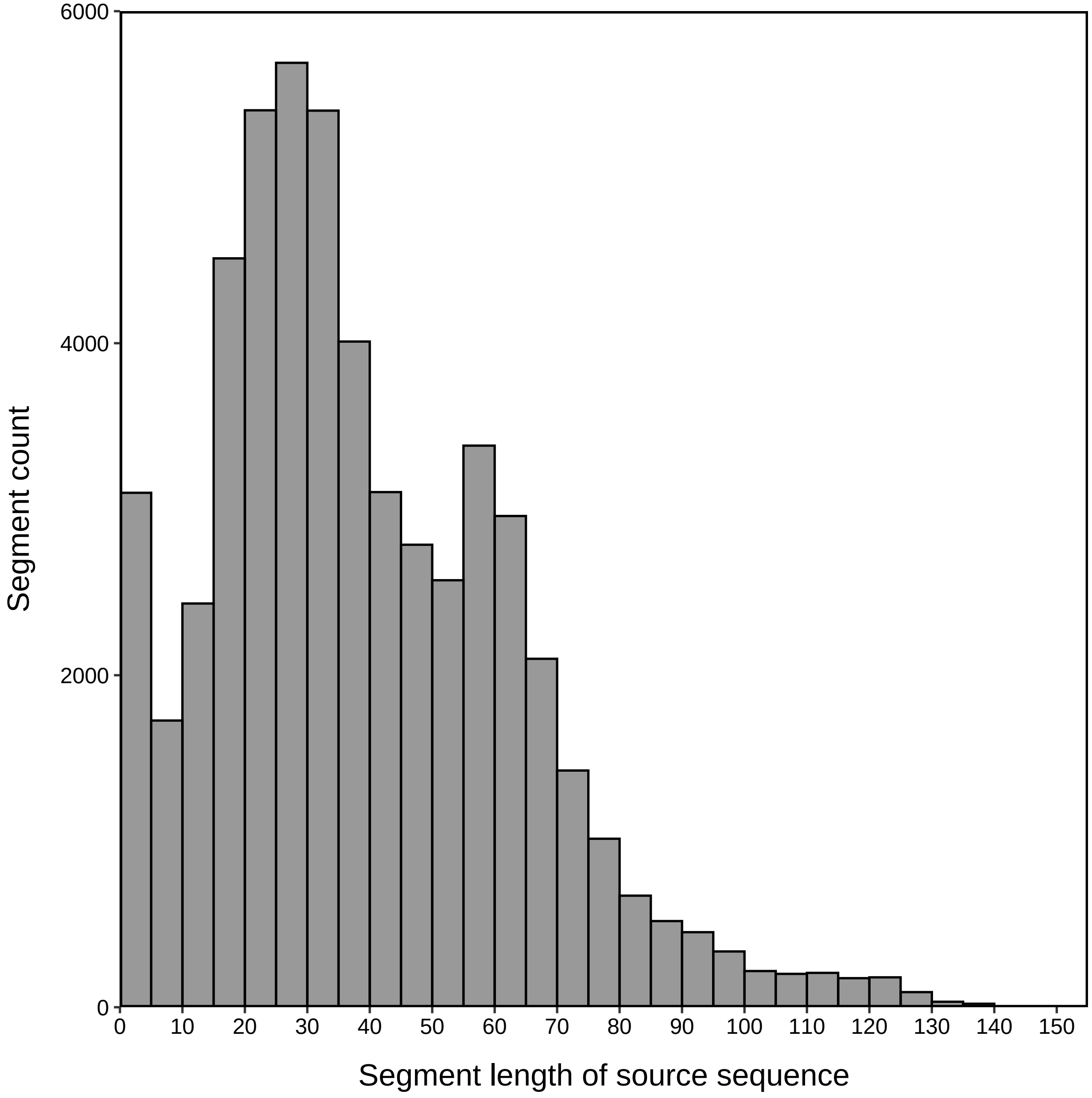

Figure S19: **Distribution of source segment length in mosaic representations of Ghana data.** There is a peak of source segments which are less than 5AA, which appear to be the artifacts of the JHMM method.

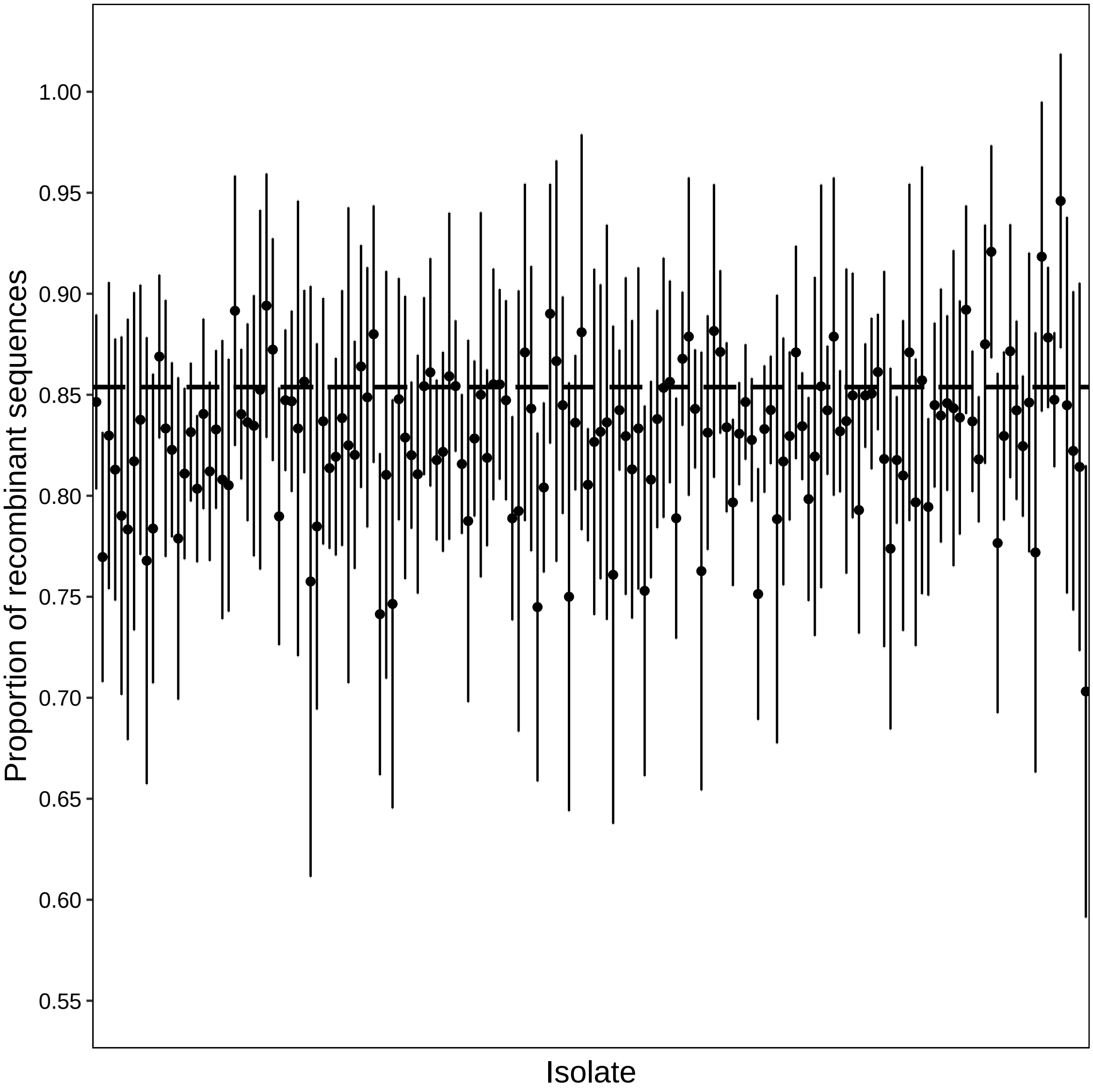

Figure S20: **Proportions (and 95% confidence intervals) of recombinants for each isolate.** The horizontal dashed line displays the overall proportion of recombinant sequences in the entire dataset.

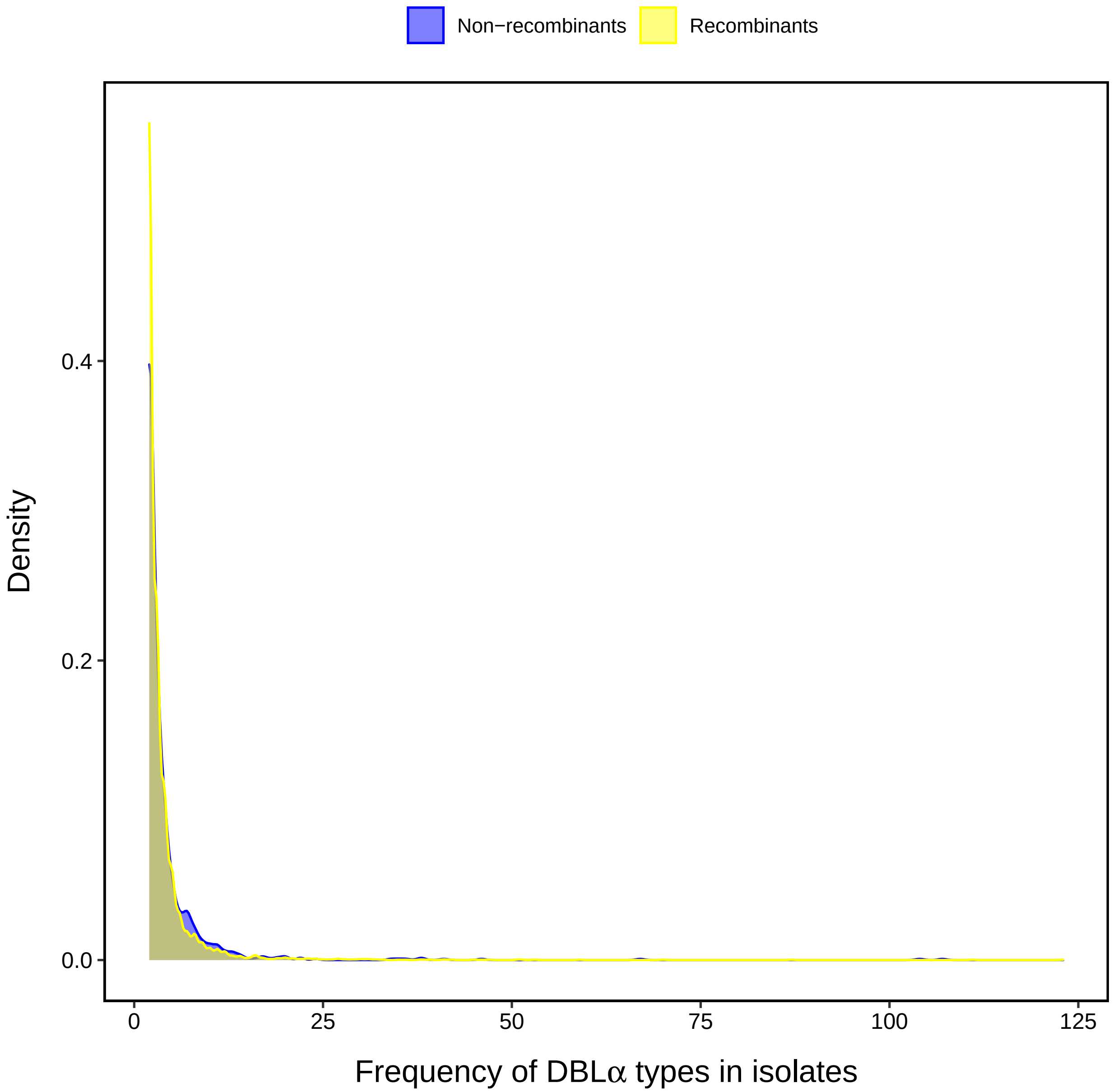

Figure S21: **Frequency of DBLα types in the isolates of the Ghana dataset.**

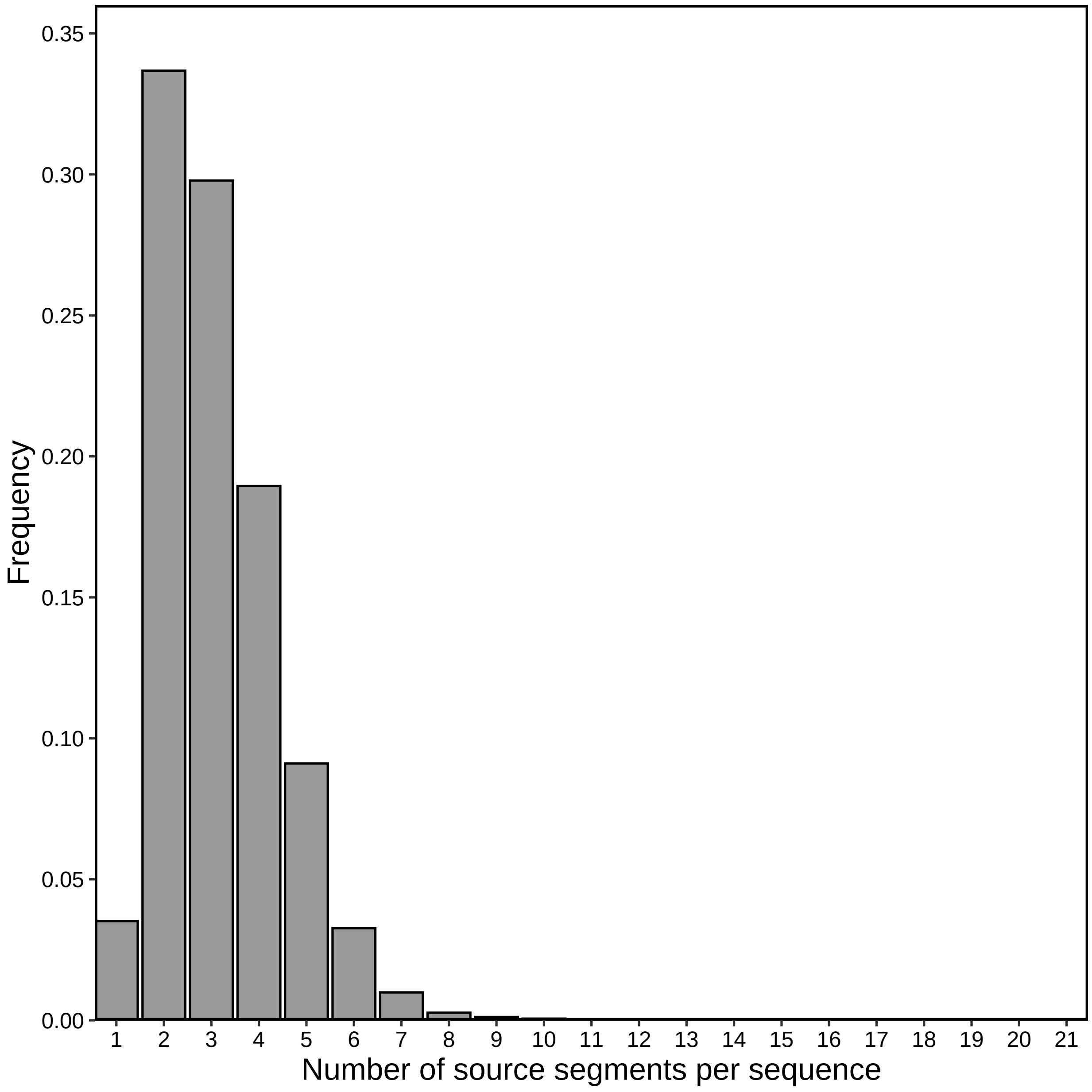

Figure S22: **Distribution of source segment count from the JHMM output in the Ghana data.**

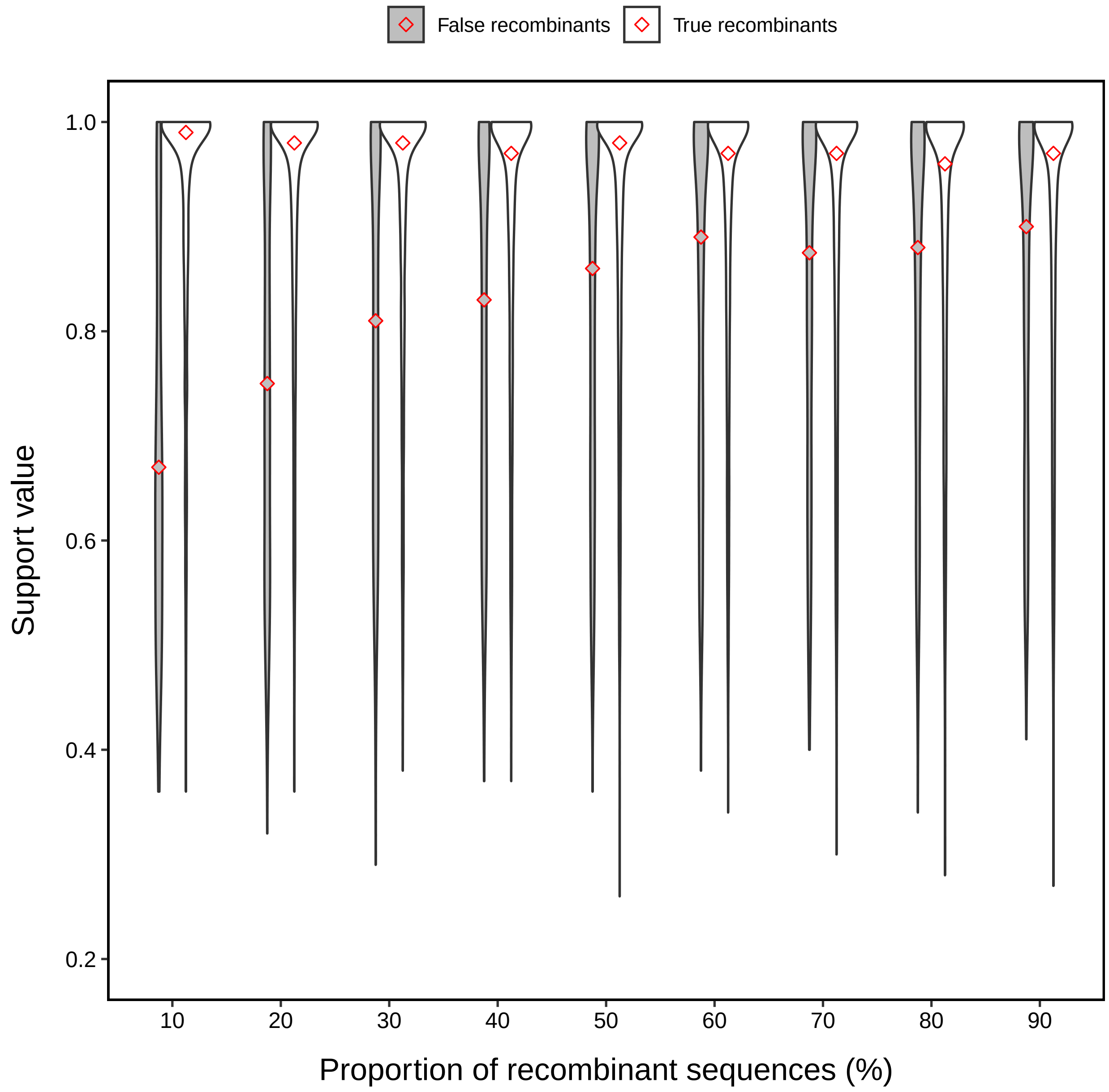

Figure S23: **Distribution of support values for varying proportions of recombinant sequences.** Red points represent the median of support values (same hereinafter).

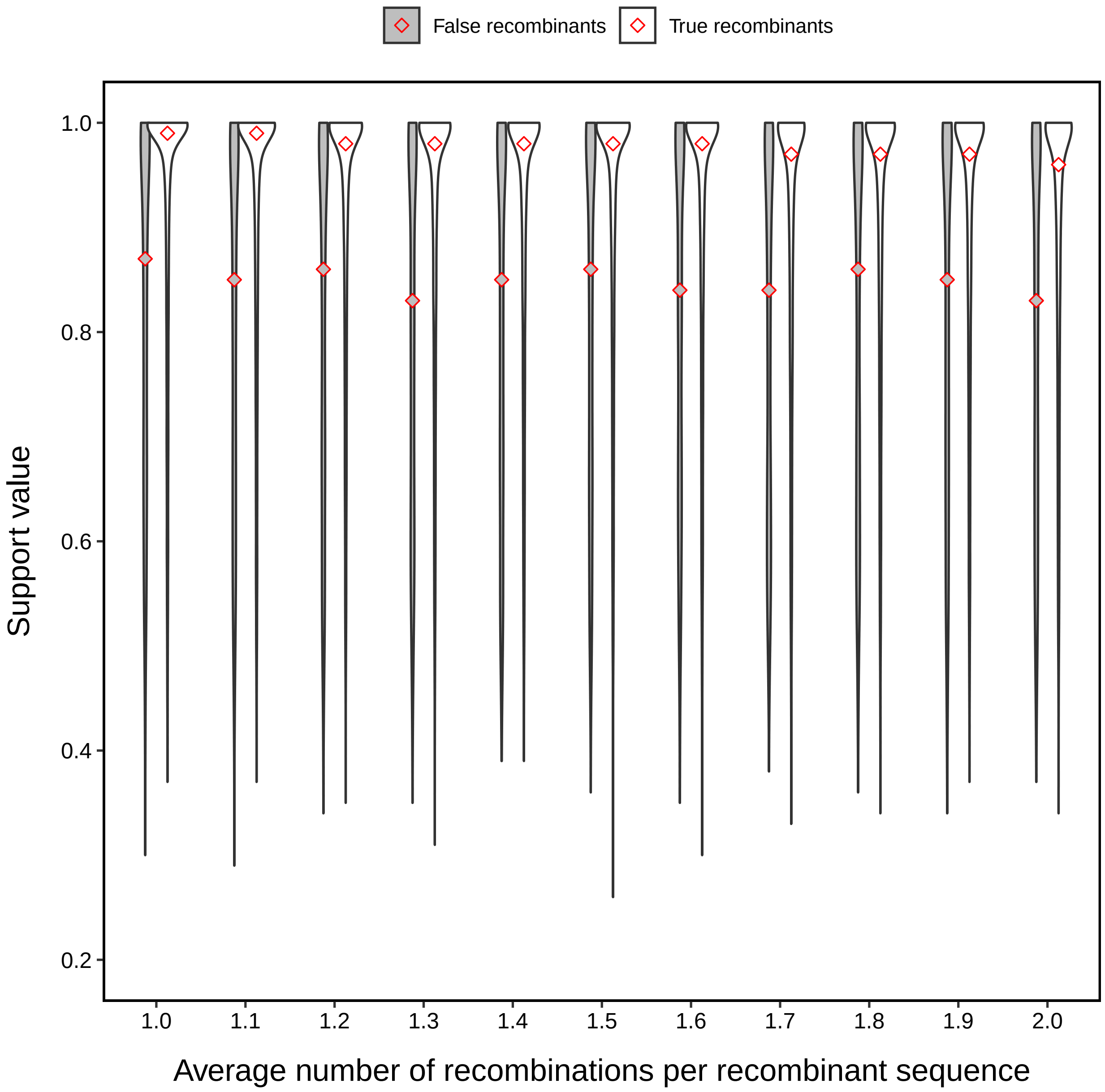

Figure S24: **Distribution of support values for varying numbers of recombinations per recombinant sequence.**

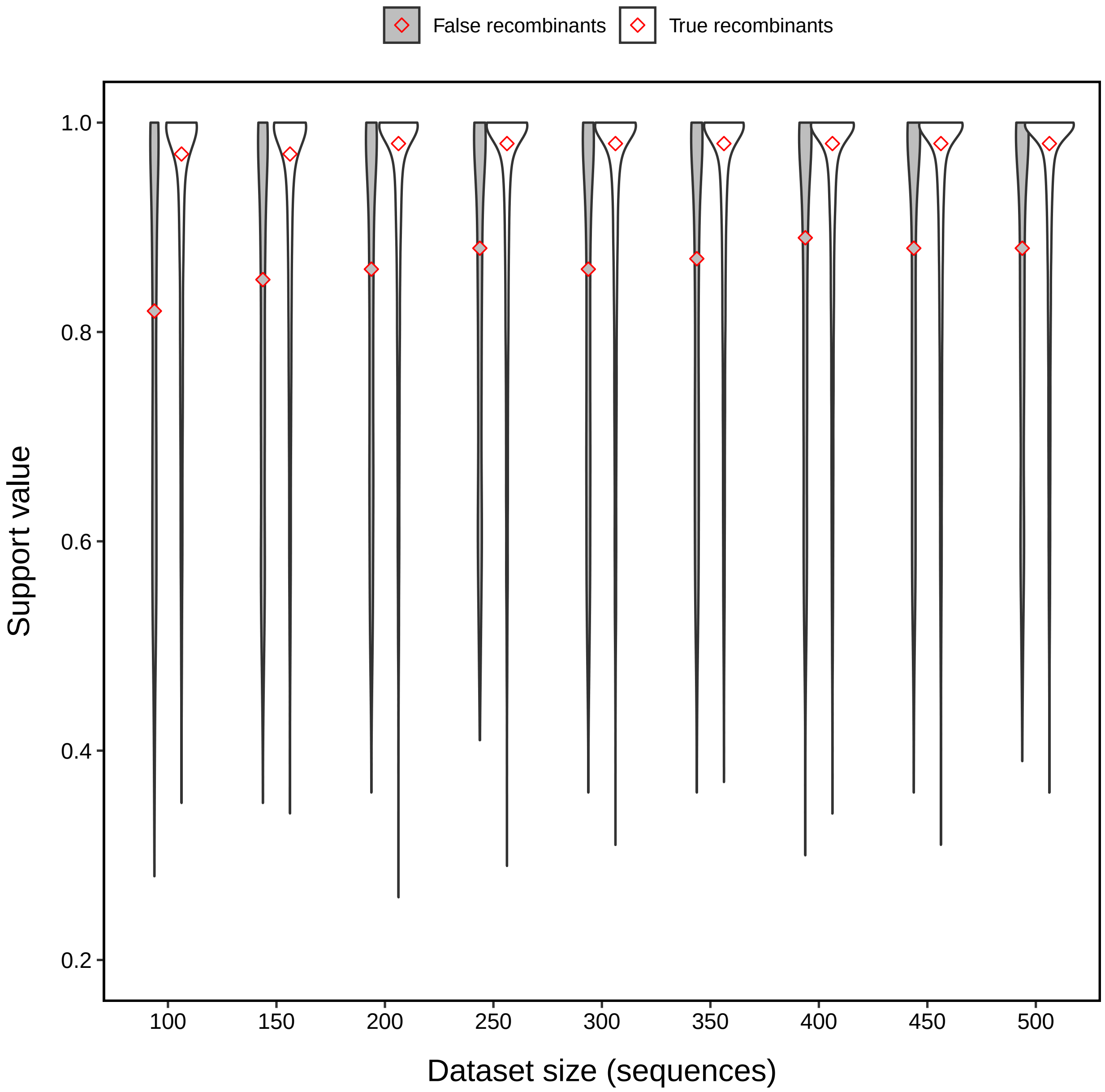

Figure S25: **Distribution of support values for varying dataset size.**

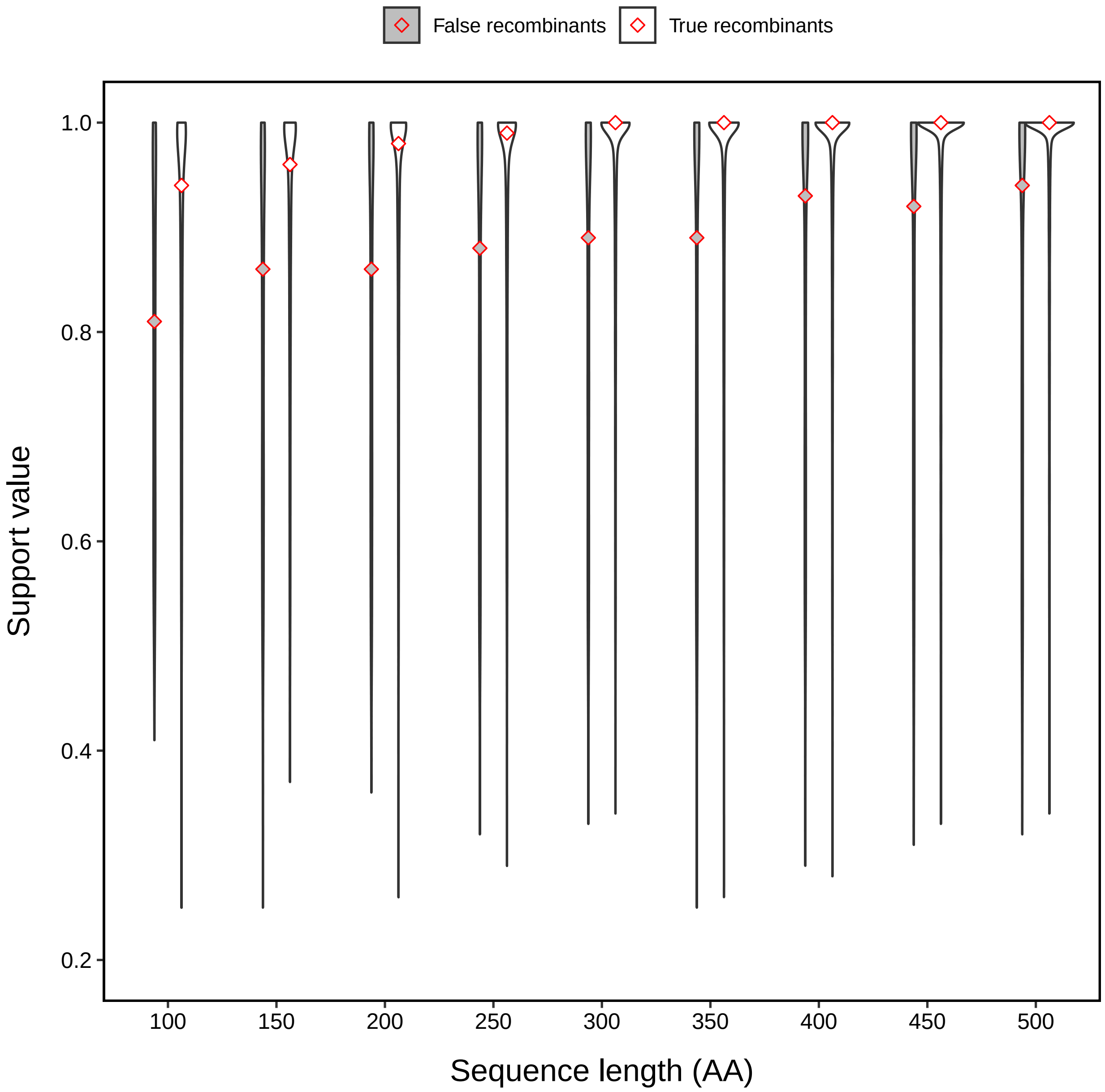

Figure S26: **Distribution of support values for varying sequence length.**

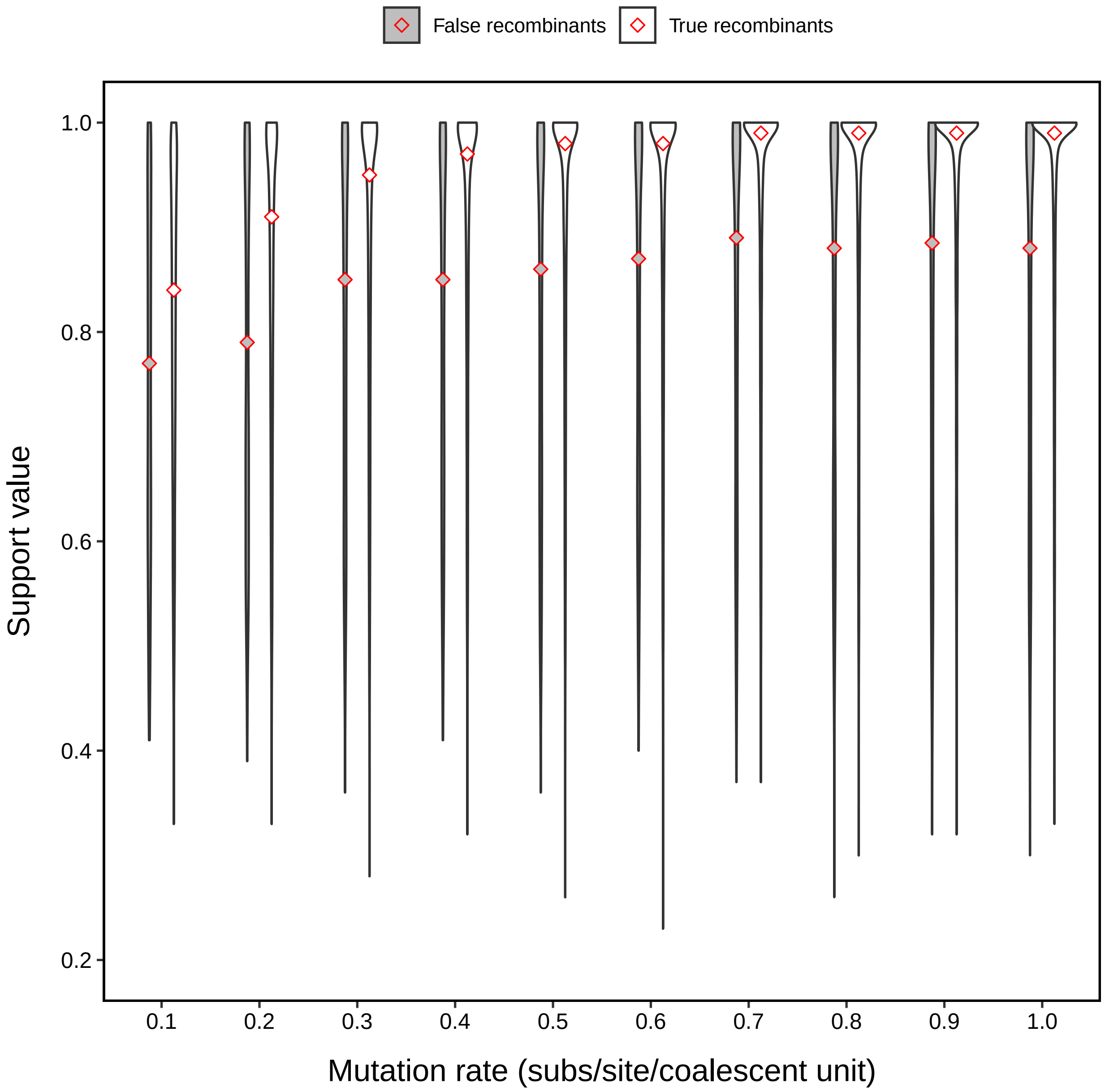

Figure S27: **Distribution of support values for varying mutation rate.**

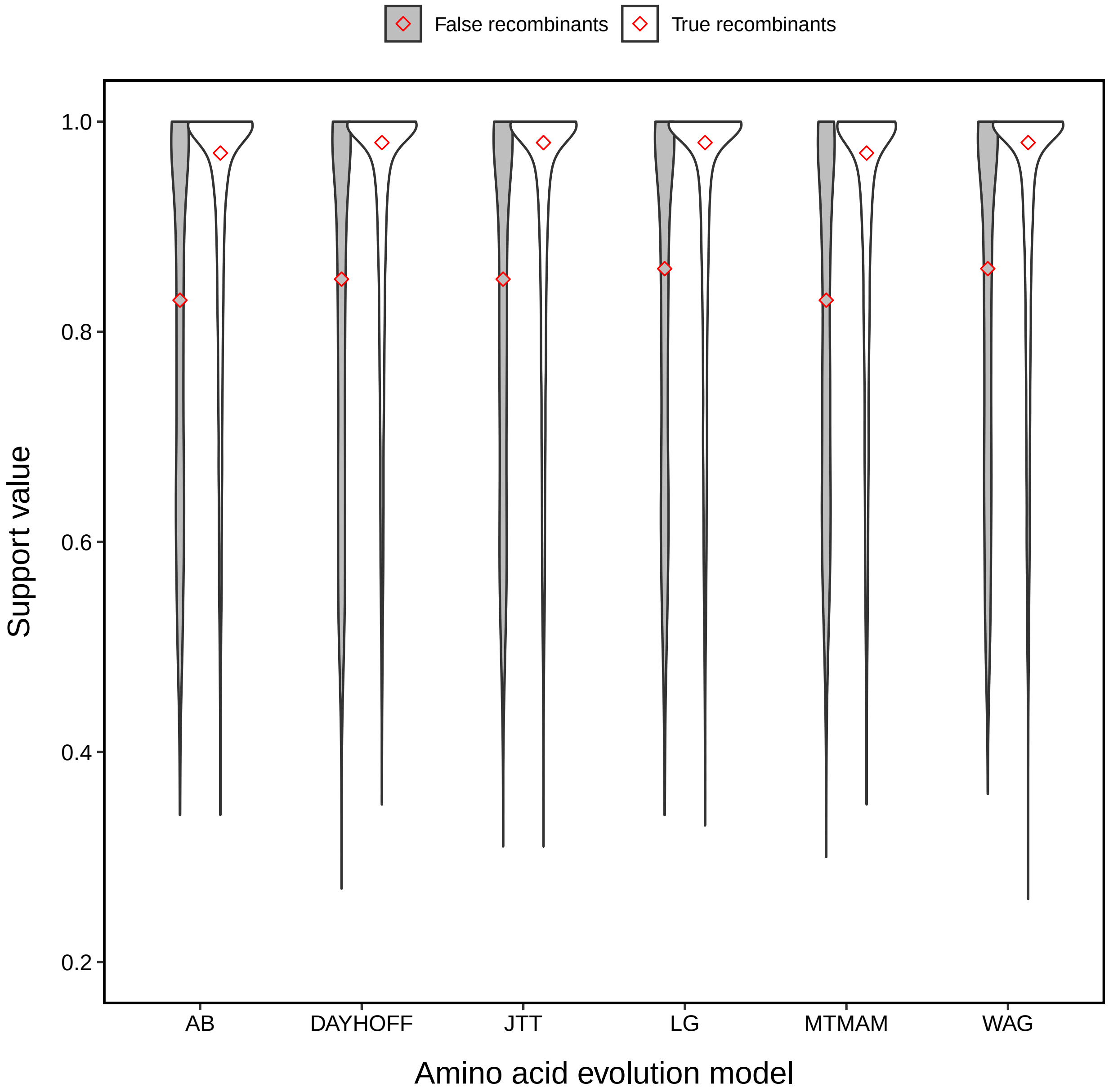

Figure S28: **Distribution of support values for different models of amino acid evolution.**

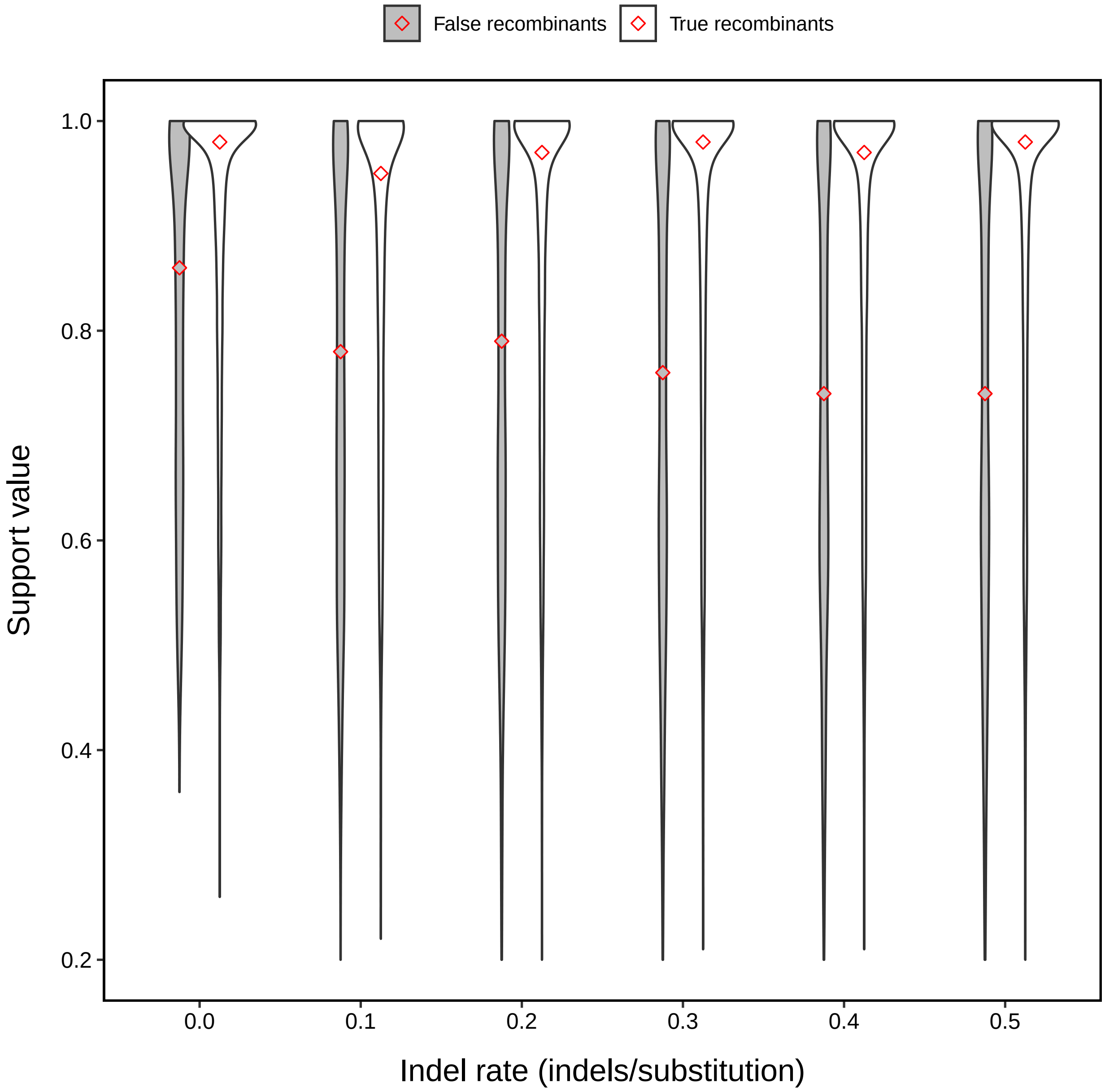

Figure S29: **Distributions of support values for varying indel rate.**

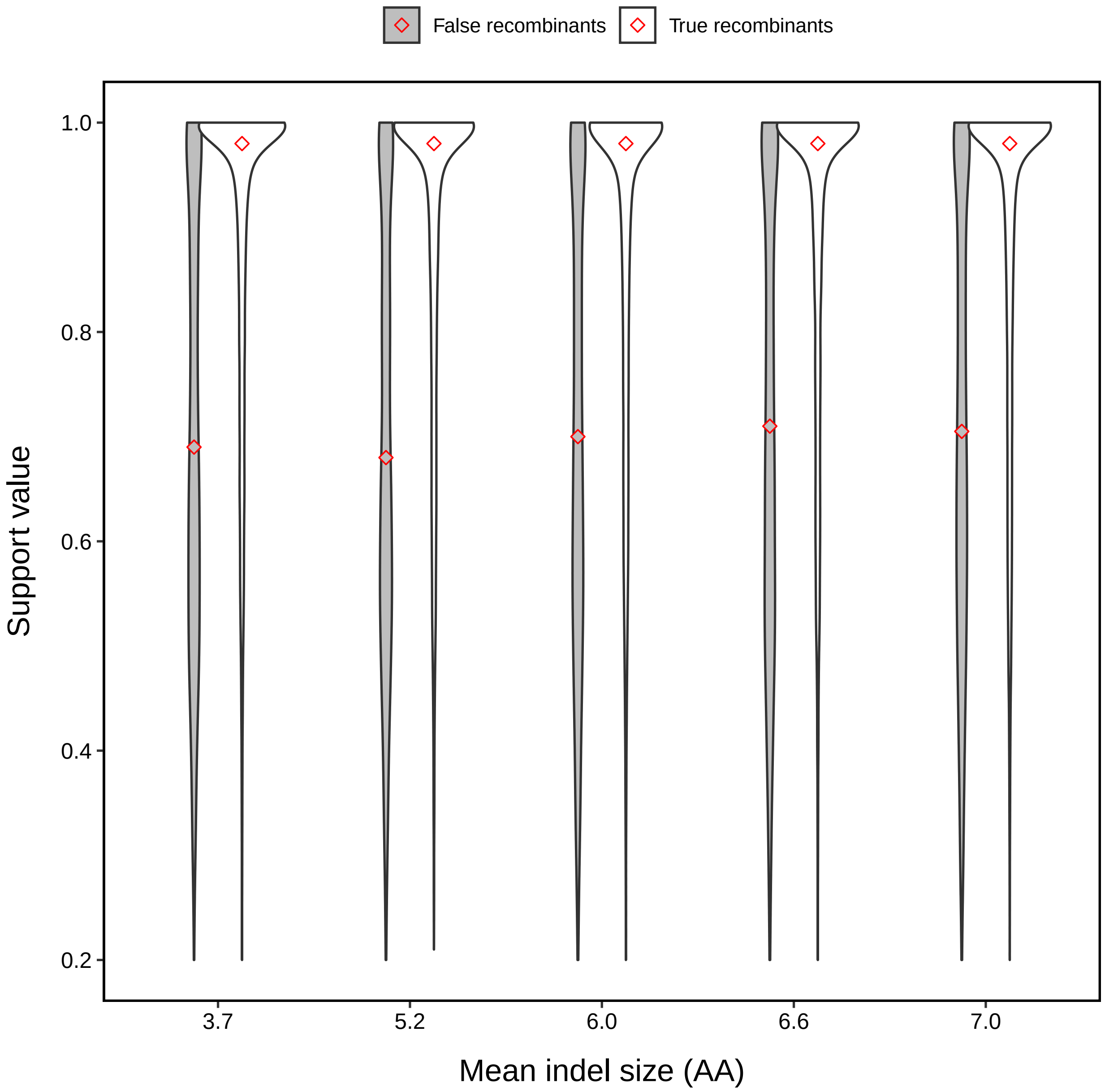

Figure S30: **Distributions of support values for varying indel size.**

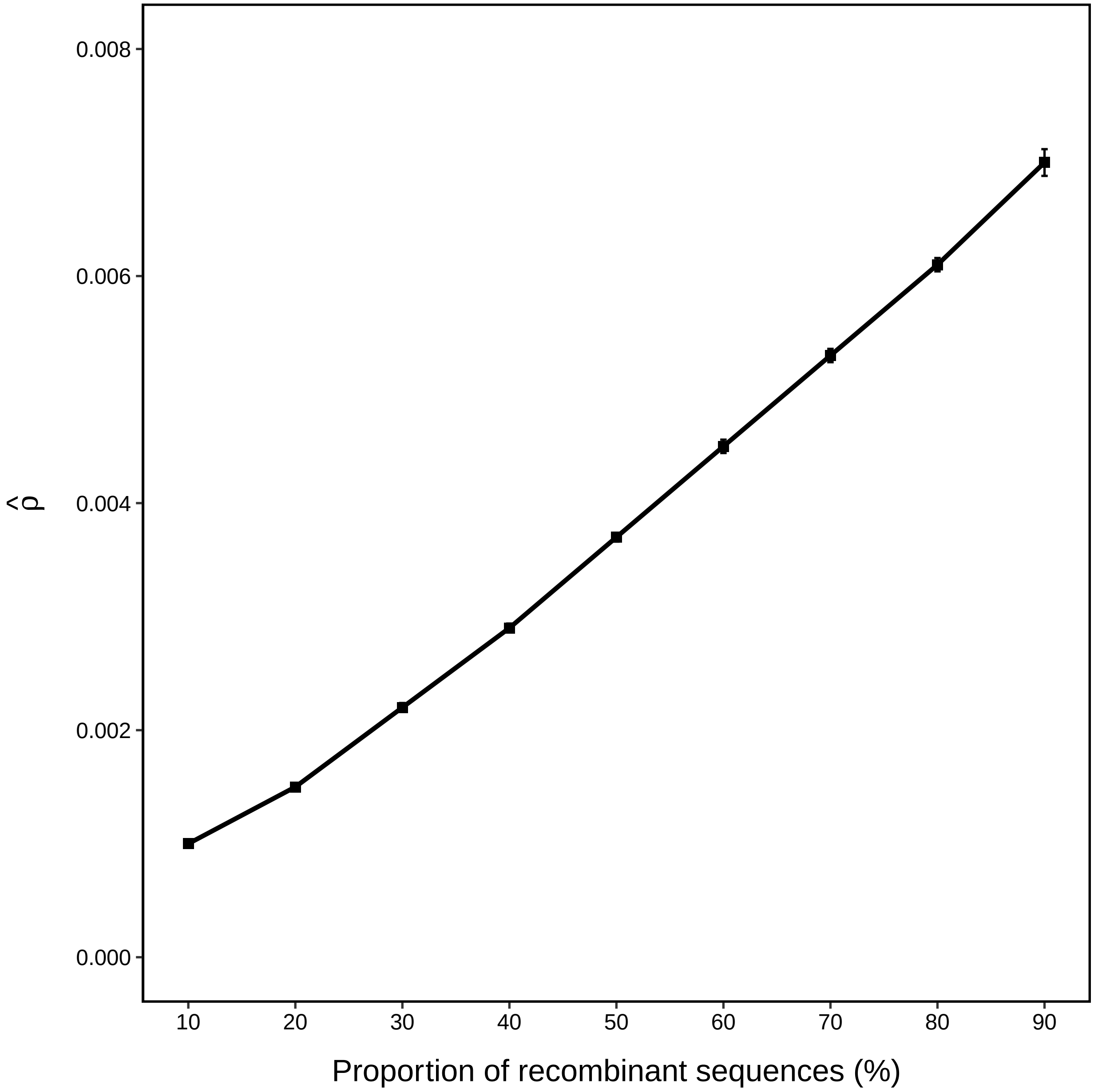

Figure S31: **Estimated ρ (and 95% CI) for varying proportions of recombinant sequences.** Some CIs are too short to be visible (similarly for Figures S32–S34). $\hat{\rho}$ appears to grow linearly with the proportion of recombinant sequences, as expected.

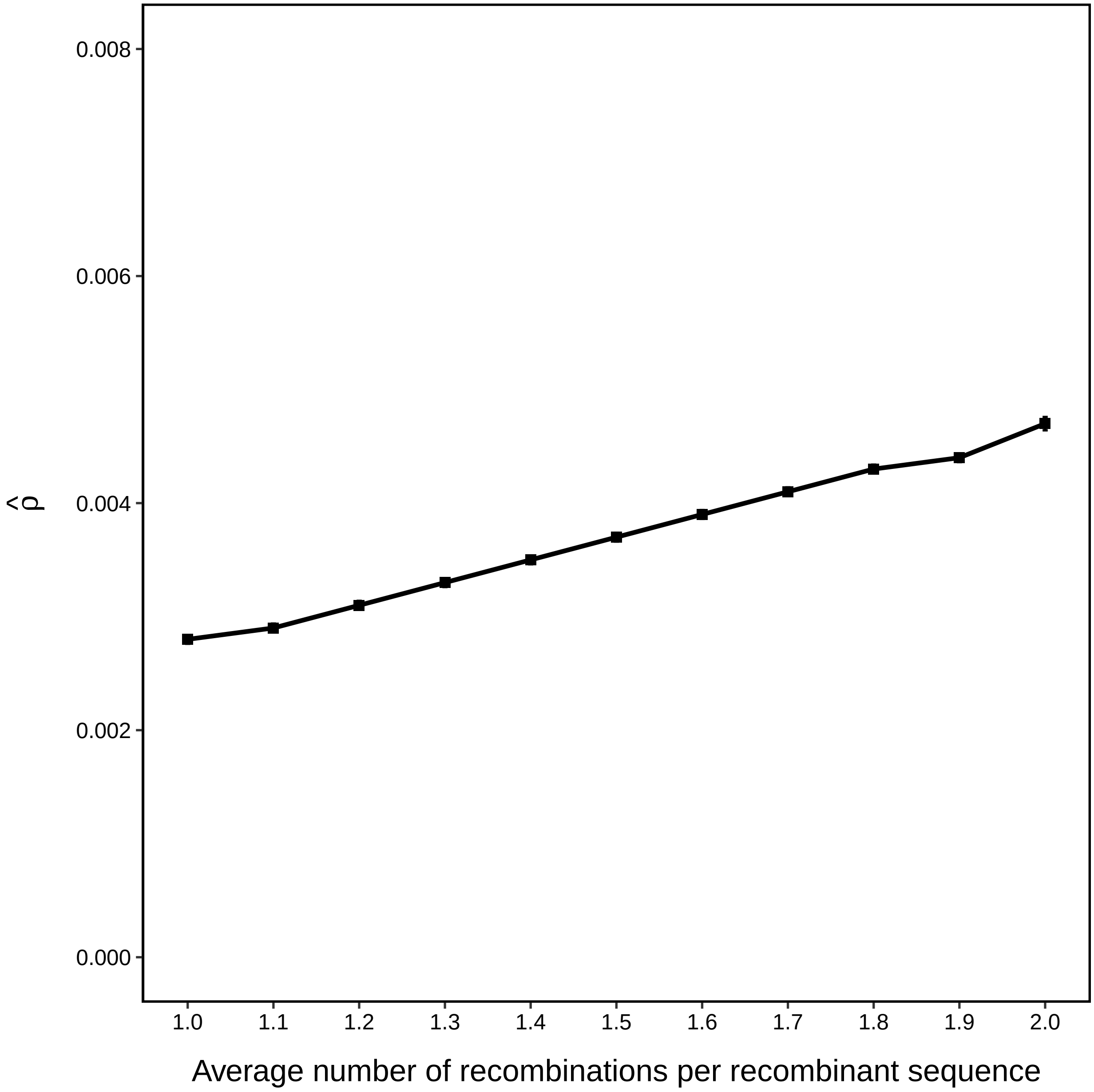

Figure S32: **Estimated ρ (and 95% CI) for varying number of recombinations per recombinant sequence.** $\hat{\rho}$ appears to grow linearly with the number of recombinants per sequence, as expected.

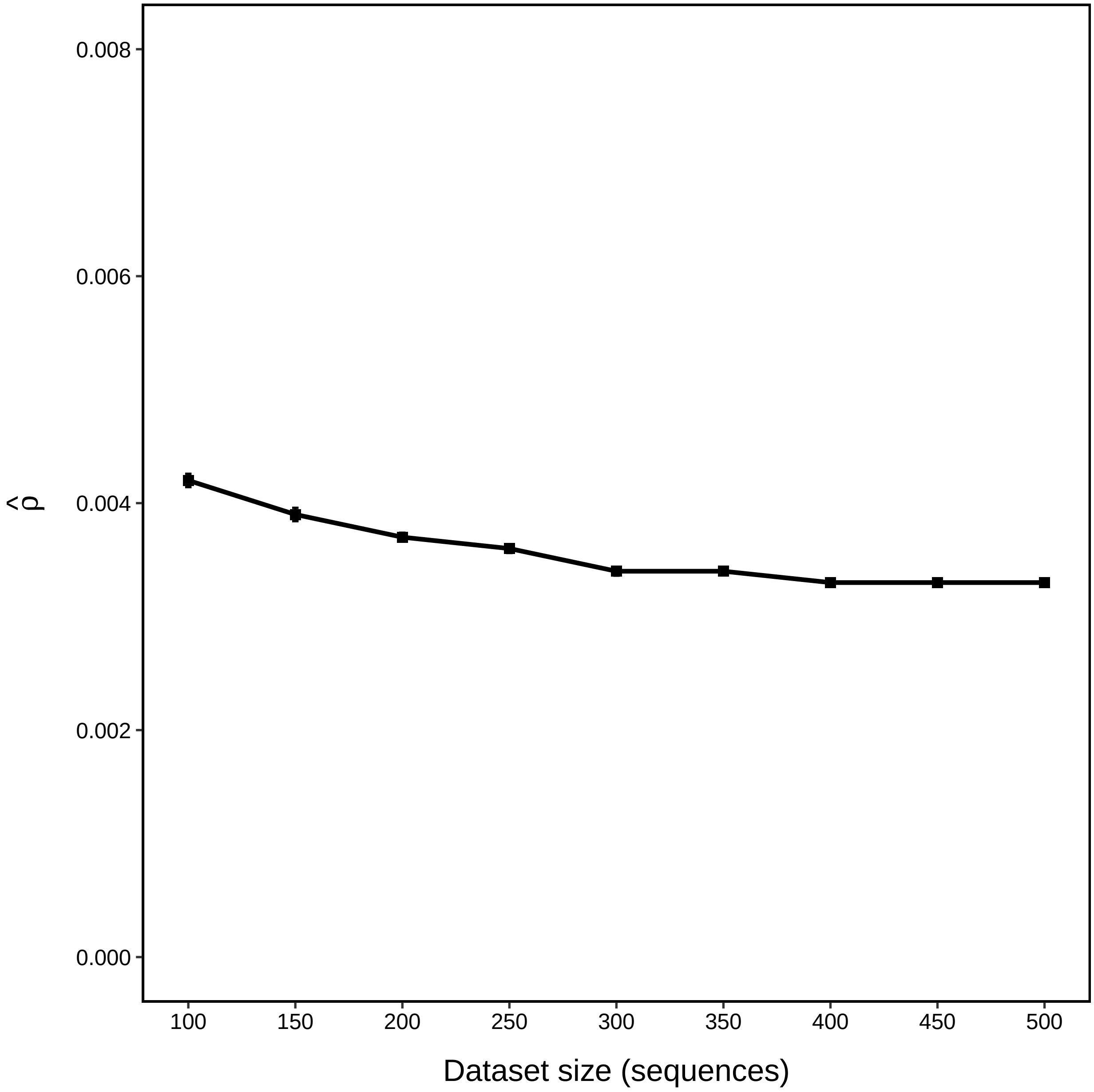

Figure S33: **Estimated ρ (and 95% CI) for varying dataset size.** $\hat{\rho}$ decreases slightly with increasing dataset size, although the recombination rate remains constant.

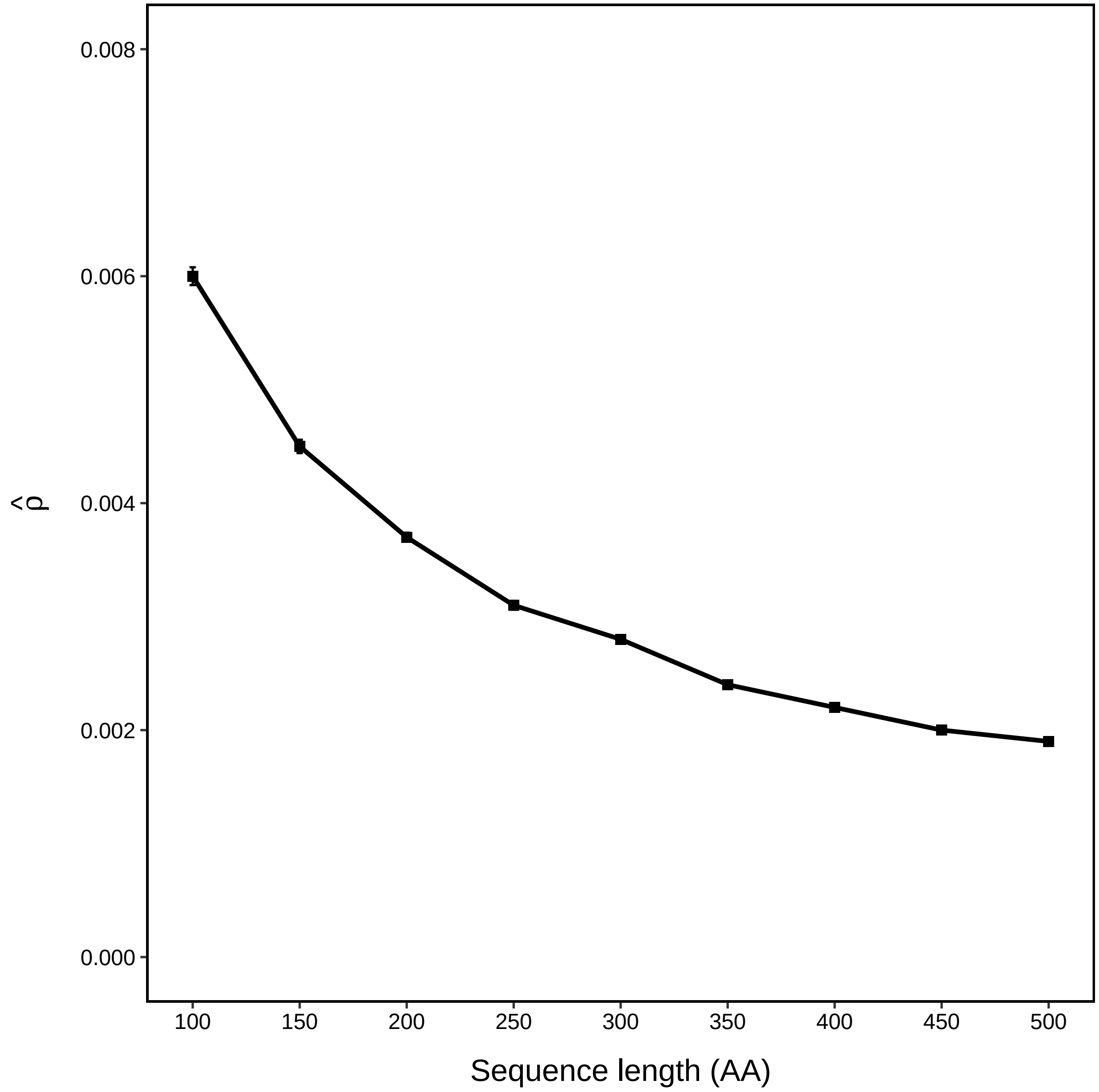

Figure S34: **Estimated ρ (and 95% CI) for varying sequence length.** $\hat{\rho}$ decreases in inverse proportion to the sequence length, as expected.

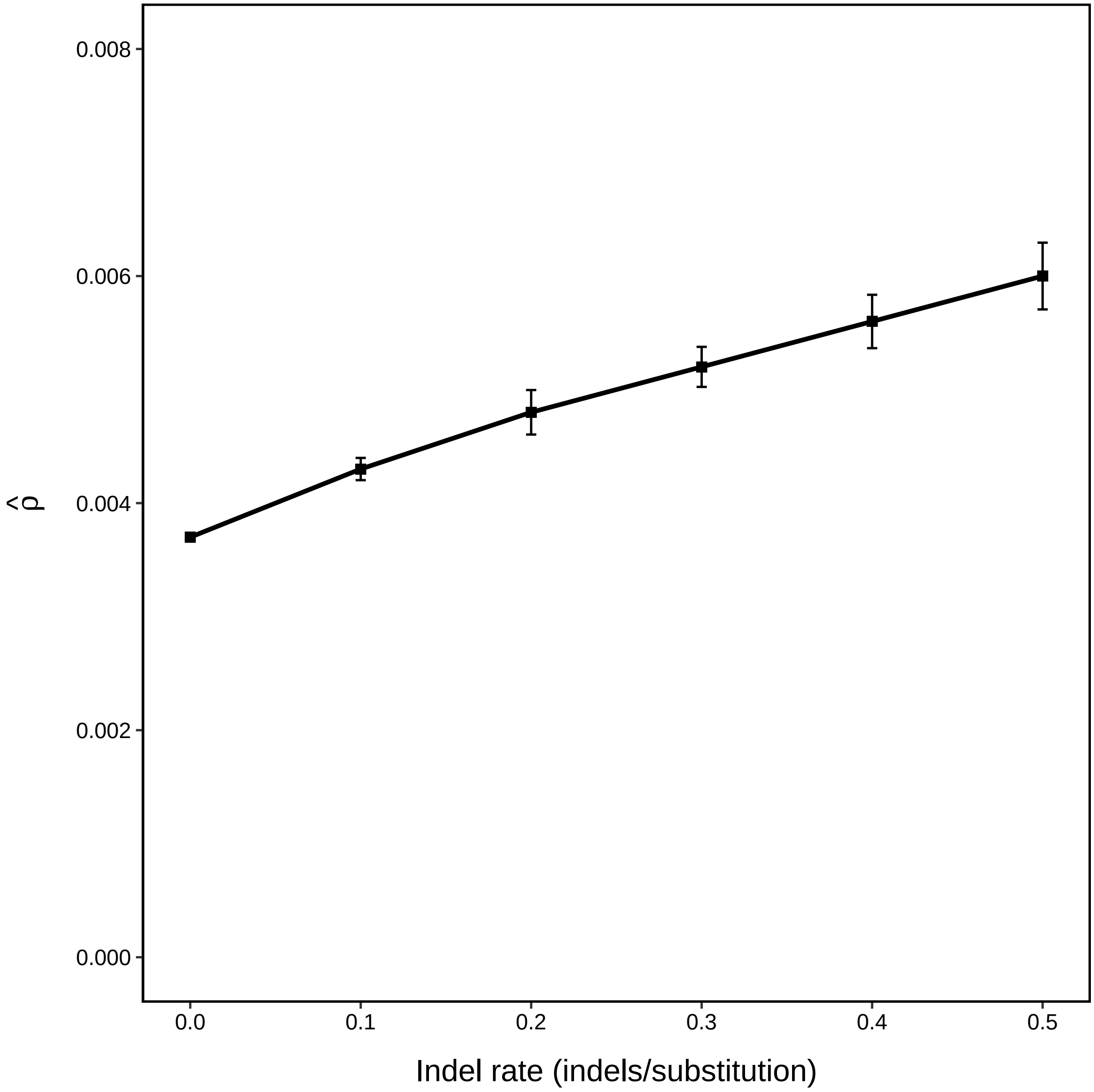

Figure S35: **Estimated ρ (and 95% CI) for varying indel rate.** There is a moderate increase in $\hat{\rho}$ as indel rate increases. This is unsurprising, as some of indel events are mistaken for recombinations, distorting the inference of the recombination rate.

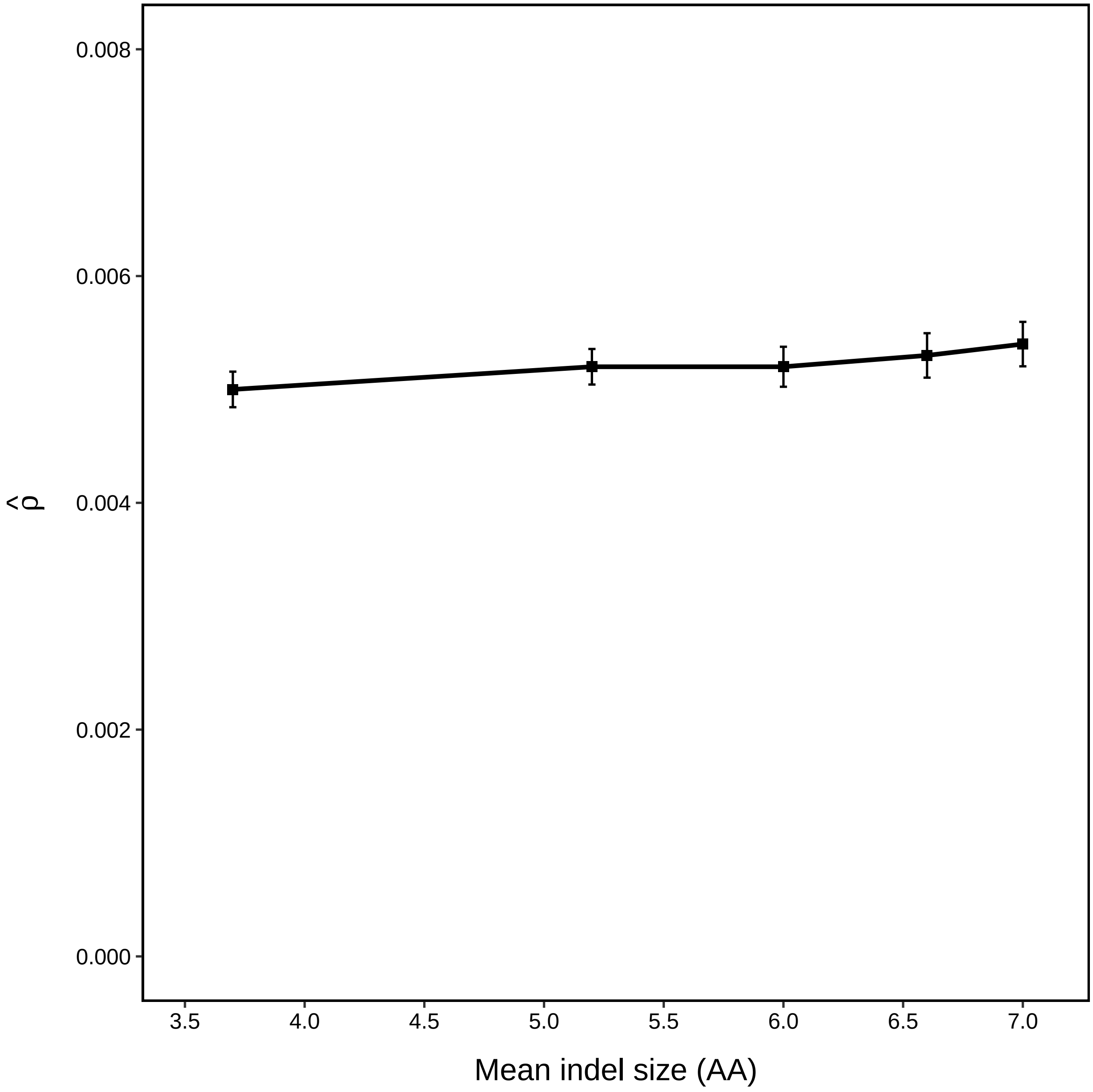

Figure S36: **Estimated ρ (and 95% CI) for varying indel size.** Indel size (but constant indel rate) does not appear to have a drastic effect on the estimated ρ.

References

Barry, A. E., Leliwa-Sytek, A., Tavul, L., Imrie, H., Migot-Nabias, F., Brown, S. M., McVean, G. A., and Day, K. P. (2007). Population genomics of the immune evasion (var) genes of *Plasmodium falciparum*. *PLOS Pathog.*, **3**(3), e34.

Boni, M. F., Posada, D., and Feldman, M. W. (2007). An exact nonparametric method for inferring mosaic structure in sequence triplets. *Genetics*, **176**(2), 1035–1047.

Dayhoff, M., Schwartz, R., and Orcutt, B. (1978). Chapter 22: A model of evolutionary change in proteins. In *Atlas of Protein Sequence and Structure*, volume 5, pages 345–352. National Biomedical Research Foundation Silver Spring MD.

Edgar, R. C. (2004). Muscle: a multiple sequence alignment method with reduced time and space complexity. *BMC Bioinform.*, **5**(1), 1–19.

Edgar, R. C. (2010). Search and clustering orders of magnitude faster than blast. *Bioinformatics*, **26**(19), 2460–2461.

Felsenstein, J. (1981). Evolutionary trees from dna sequences: a maximum likelihood approach. *J. Mol. Evol.*, **17**(6), 368–376.

Gibbs, M. J., Armstrong, J. S., and Gibbs, A. J. (2000). Sister-Scanning: a Monte Carlo procedure for assessing signals in recombinant sequences. *Bioinformatics*, **16**(7), 573–582.

Huson, D. H., Rupp, R., and Scornavacca, C. (2010). *Phylogenetic networks: concepts, algorithms and applications*. Cambridge University Press.

Jones, D., Taylor, W., and Thornton, J. (1994). A mutation data matrix for transmembrane proteins. *FEBS Letters*, **339**(3), 269–275.

Kingman, J. F. (1982). On the genealogy of large populations. *J. Appl. Probab.*, **19**(A), 27–43.

Lam, H. M., Ratmann, O., and Boni, M. F. (2018). Improved algorithmic complexity for the 3seq recombination detection algorithm. *Mol. Biol. Evol.*, **35**(1), 247–251.

Le, S. Q. and Gascuel, O. (2008). An improved general amino acid replacement matrix. *Mol. Biol. Evol.*, **25**(7), 1307–1320.

Martin, D. and Rybicki, E. (2000). RDP: detection of recombination amongst aligned sequences. *Bioinformatics*, **16**(6), 562–563.

Martin, D. P., Varsani, A., Roumagnac, P., Botha, G., Maslamoney, S., Schwab, T., Kelz, Z., Kumar, V., and Murrell, B. (2020). Rdp5: A computer program for analysing recombination in, and removing signals of recombination from, nucleotide sequence datasets. *Virus Evol.*

Mirsky, A., Kazandjian, L., and Anisimova, M. (2015). Antibody-specific model of amino acid substitution for immunological inferences from alignments of antibody sequences. *Mol. Biol. Evol.*, **32**(3), 806–819.

Padidam, M., Sawyer, S., and Fauquet, C. M. (1999). Possible emergence of new geminiviruses by frequent recombination. *Virol. J.*, **265**(2), 218–225.

Pardi, F. and Scornavacca, C. (2015). Reconstructible phylogenetic networks: do not distinguish the indistinguishable. *PLoS Comput. Biol.*, **11**(4).

Posada, D. and Crandall, K. A. (2001). Evaluation of methods for detecting recombination from DNA sequences: computer simulations. *Proc. Natl. Acad. Sci.*, **98**(24), 13757–13762.

Rask, T. S., Hansen, D. A., Theander, T. G., Pedersen, A. G., and Lavstsen, T. (2010). *Plasmodium falciparum* erythrocyte membrane protein 1 diversity in seven genomes–divide and conquer. *PLoS Comput. Biol.*, **6**(9), e1000933.

Rorick, M. M., Rask, T. S., Baskerville, E. B., Day, K. P., and Pascual, M. (2013). Homology blocks of *Plasmodium falciparum* var genes and clinically distinct forms of severe malaria in a local population. *BMC Microbiol.*, **13**(1), 244.

Rorick, M. M., Artzy-Randrup, Y., Ruybal-Pesántez, S., Tiedje, K. E., Rask, T. S., Oduro, A., Ghansah, A., Koram, K., Day, K. P., and Pascual, M. (2018). Signatures of competition and strain structure within the major blood-stage antigen of *Plasmodium falciparum* in a local community in ghana. *Ecol. Evol.*, **8**(7), 3574–3588.

Ruybal-Pesántez, S., Tiedje, K. E., Rorick, M. M., Amenga-Etego, L., Ghansah, A., Oduro, A. R., Koram, K. A., and Day, K. P. (2017a). Lack of geospatial population structure yet significant linkage disequilibrium in the reservoir of *Plasmodium falciparum* in Bongo District, Ghana. *Am. J. Trop. Med. Hyg.*, **97**(4), 1180–1189.

Ruybal-Pesántez, S., Tiedje, K. E., Tonkin-Hill, G., Rask, T. S., Kamya, M. R., Greenhouse, B., Dorsey, G., Duffy, M. F., and Day, K. P. (2017b). Population genomics of virulence genes of *Plasmodium falciparum* in clinical isolates from Uganda. *Sci. Rep.*, **7**(1), 11810.

Smith, J. M. (1992). Analyzing the mosaic structure of genes. *J. Mol. Evol.*, **34**(2), 126–129.

Tiedje, K. E., Oduro, A. R., Agongo, G., Anyorigiya, T., Azongo, D., Awine, T., Ghansah, A., Pascual, M., Koram, K. A., and Day, K. P. (2017). Seasonal variation in the epidemiology of asymptomatic *Plasmodium falciparum* infections across two catchment areas in Bongo District, Ghana. *Am. J. Trop. Med. Hyg.*, **97**(1), 199–212.

Tonkin-Hill, G., Ruybal-Pesántez, S., Tiedje, K. E., Rougeron, V., Duffy, M. F., Zakeri, S., Pumpaibool, T., Harnyuttanakorn, P., Branch, O. H., Ruiz-Mesía, L., *et al.* (2021). Evolutionary analyses of the major variant surface antigen-encoding genes reveal population structure of *Plasmodium falciparum* within and between continents. *PLoS Genet.*, **17**(2), e1009269.

Yang, Z., Nielsen, R., and Hasegawa, M. (1998). Models of amino acid substitution and applications to mitochondrial protein evolution. *Mol. Biol. Evol.*, **15**(12), 1600–1611.

Zilversmit, M. M., Chase, E. K., Chen, D. S., Awadalla, P., Day, K. P., and McVean, G. (2013). Hypervariable antigen genes in malaria have ancient roots. *BMC Evol. Biol.*, **13**(1), 110.
